# Genome-Wide Detection of Gene Coexpression Domains Showing Linkage to Regions Enriched with Polymorphic Retrotransposons in Recombinant Inbred Mouse Strains

**DOI:** 10.1534/g3.113.005546

**Published:** 2013-04-01

**Authors:** Marie-Pier Scott-Boyer, Christian F. Deschepper

**Affiliations:** Cardiovascular Biology Research Unit, Institut de recherches cliniques de Montréal (IRCM) and Université de Montréal, Montréal, Québec, H2W 1R7, Canada

**Keywords:** genetics of gene expression, quantitative trait loci, clustering of coexpressed genes, transposable elements, structural variants

## Abstract

Although gene coexpression domains have been reported in most eukaryotic organisms, data available to date suggest that coexpression rarely concerns more than doublets or triplets of adjacent genes in mammals. Using expression data from hearts of mice from the panel of AxB/BxA recombinant inbred mice, we detected (according to window sizes) 42−53 loci linked to the expression levels of clusters of three or more neighboring genes. These loci thus formed “*cis*-expression quantitative trait loci (eQTL) clusters” because their position matched that of the genes whose expression was linked to the loci. Compared with matching control regions, genes contained within *cis*-eQTL clusters showed much greater levels of coexpression. Corresponding regions showed: (1) a greater abundance of polymorphic elements (mostly short interspersed element retrotransposons), and (2) significant enrichment for the motifs of binding sites for various transcription factors, with binding sites for the chromatin-organizing CCCTC-binding factor showing the greatest levels of enrichment in polymorphic short interspersed elements. Similar *cis*-eQTL clusters also were detected when we used data obtained with several tissues from BxD recombinant inbred mice. In addition to strengthening the evidence for gene expression domains in mammalian genomes, our data suggest a possible mechanism whereby noncoding polymorphisms could affect the coordinate expression of several neighboring genes.

A significant component of gene expression variability in populations is due to variations in the DNA sequence (Schadt *et al.* 2003). Accordingly, genetic mapping studies have led to the identification of quantitative trait loci (QTL) linked to the expression levels of particular genes within cells and/or tissues (Göring *et al.* 2007). When the expression of a given gene associates with a genetic polymorphism that maps close to that gene’s locus, the corresponding “expression QTL” (eQTL) is identified as a proximal eQTL (also called “*cis*-eQTL”). In other cases, when the expression level of a gene associates with a locus clearly distinct from that of the gene itself, it is defined as a distal eQTL (also called “*trans*-eQTL”). In the case of *trans*-eQTLs, it has been observed that a single genetic locus can show linkage to the abundance of the mRNA transcript of several genes across the genome, forming so-called *trans*-eQTL “hotspots” or “bands” (Grieve *et al.* 2008; Wu *et al.* 2008). One example would, for instance, be that of a *cis*-eQTL regulating the expression of a transcription factor, which in turns regulates the expression of many other genes belonging to a hotspot of *trans*-eQTLs.

Contrary to *trans*-eQTLs, investigators typically associate *cis*-eQTLs with the expression level of just one gene. The premise is that the *cis*-acting polymorphism that is located in close proximity to the gene is likely to affect the regulatory machinery of that same gene and that machinery is unlikely to be shared by genes other than the ones that are immediately adjacent. Nonetheless, if the genome contained features that could influence the expression of several neighboring (and not necessary immediately adjacent) genes, one consequence from a genetic standpoint would be the clustering of several *cis*-eQTLs within a narrow genetic interval.

Although clustering of *cis*-eQTLs has not been reported yet, there is evidence that gene coexpression domains exist in several eukaryotic organisms (Michalak 2008; Elizondo *et al.* 2009). For instance, approximately 20% of the genes in Drosophila are arranged into clusters of similarly expressed genes, with the clusters spanning intervals from 20 to 200 kb and containing 10 to 30 genes each (Spellman and Rubin 2002). In mammals, coexpressed genes have been reported to cluster both at either short-range (1 Mb) or long-range (>10 Mb) levels (Woo *et al.* 2010). One particular case of short-range coexpression clusters concerns that of conserved clusters of paralogous genes arising from tandem duplication (such as, for instance, Hox, globin, and major histocompatibility complex genes). Beyond these paralogous clusters, it was reported in humans that the overall level of coexpression of genes was greater than expected by chance when the genes are located within distances smaller than 1 Mb, although the level of expression did not exceed that of more distant genes by a very large margin (Lercher *et al.* 2002). Likewise, in other studies reporting on the clustering of co-expressed genes in mammals, it was found that coexpression rarely concerned more than doublets or triplets of immediately adjacent genes (Sémon and Duret 2006; Purmann *et al.* 2007). In such cases, coexpression is generally believed to derive from the sharing of one regulatory element by adjacent genes. Nonetheless, clusters containing on average two to six coordinately regulated genes within 1-Mb intervals have been observed under special circumstances, such as in fibroblasts during replicative senescence (Zhang *et al.* 2003).

One limitation of many studies to date is that they have been not been performed within the framework of panels of individuals (or strains) with well-characterized genetic backgrounds. If “short-range” clustering of coexpressed genes could derive from physical elements in the genome, the impact of the latter would be easier to detect in situations in which they are polymorphic, such as in animals from genetic crosses. Moreover, because clusters of *cis*-eQTLs would all map to precise genomic regions, further analysis of these regions might reveal the nature of the polymorphisms associated with coordinate changes in gene expression.

To complement previous genetic studies reporting on QTL linked to cardiac morphologic characteristics, we used Illumina microarrays to obtain the profiles of cardiac gene expression in a panel of 24 mouse recombinant inbred strains (RIS). When performing linkage analysis to detect eQTLs for all detected genes, we observed several instances in which three or more *cis*-eQTLs clustered within small genomic intervals. Because such clustering of *cis*-eQTLs had not been reported previously, we used our dataset to analyze the characteristics of corresponding regions in a more systematic fashion. Likewise, to test to which extent this observation could be generalized to other tissues and/or crosses, we compared our findings with those obtained in other tissues from either the same or other mouse RIS panels. We found that clusters of *cis*-eQTLs could be detected in all the tissues from all genetic mouse crosses we tested and that coexpression of *cis*-eQTLs within these clusters reached very high levels. Further analysis of these regions revealed that they showed enrichment for particular types of structural polymorphisms.

## Materials and Methods

### Detection of eQTLs in hearts from AxB/BxA mouse RIS

The AxB/BxA mouse RIS originate from reciprocal crosses between the two parental C57BL/6 and A/J inbred strains and were derived from 20 generations of inbreeding of the F2 progeny of these two strains (Marshall *et al.* 1992). We had previously used a set of 24 RIS from that panel to detect QTLs linked to cardiac left ventricular mass (Llamas *et al.* 2007). Using the same 24 RIS, we extracted total RNA from cardiac left ventricles from four male mice for each strain and used Illumina MouseRef-8 v2.0 BeadChips to obtain the profile of gene expression in the tissues, as described previously (Llamas *et al.* 2009). The raw data were obtained by using the BeadStudio software (Illumina) and imported into the R programming environment. The data were processed and normalized using the Limma software (Smyth 2004). After filtering out genes not detected across the chips (by retaining only genes detected in more than 50% of the biologic replicates for at least one strain), we selected a set of 8725 genes for further analysis. Processed data have been submitted for public access to GeneNetwork (www.genenetwork.org; accession number GN421).

For genomic mapping, genomic DNA was extracted from spleens of all corresponding 24 RIS using the DNeasy tissue kit (QIAGEN, Mississauga, ON). All samples were hybridized at The Jackson Laboratory on the Affymetrix Mouse Diversity Array, which contains 623,124 single-nucleotide polymorphic (SNP) and 916,269 invariant genomic probes (Yang *et al.* 2009). Signal intensities were extracted from CEL files using the MouseDivGeno package (Didion *et al.* 2012), and genotyping was performed by comparing the intensity and contrast of signals in a given line with that in the parental strains (Simecek *et al.* 2011). In total, we detected 977 informative SNPs (meaning that they were polymorphic for at least one strain among all 24 strains from the panel) defining intervals averaging 2.59 ± 2.95 Mb. The average value of the r^2^ coefficient (calculated as a descriptor of linkage disequilibrium for all pairs of adjacent informative SNPs) was r^2^ = 0.8 (where 0 is the value for perfect equilibrium and 1 is the value obtained when two markers have identical information).

To detect and map eQTLs, the data were analyzed with the “R-QTL” tool, and all other statistical analyzes were performed with the statistical language R. We used a detection threshold corresponding to a “logarithm-of-the-odds” score of 3.3, as suggested previously (Lander and Kruglyak 1995). For each eQTL, we then determined whether the transcription was regulated *in cis* or *in trans* by defining *cis*-eQTLs as those whose peak eQTL was within 1 Mbp of the physical location of the corresponding gene start. Of note, artifactual detection of eQTLs might theoretically occur when polymorphic SNPs occur within sequences corresponding to the probes used by the microarray. Although the vast majority of SNPs within probes have been shown to have no significant effect on hybridization efficiency for Illumina microarrays (Schurmann *et al.* 2012), we nonetheless used data from the Sanger website to detect all high-quality (score > 100) polymorphic SNPs, compared their positions with those of all probes in the microarray (as annotated in GeneNetwork), and verified that the polymorphisms had no impact the eQTL analysis.

### Origin of datasets

Lists of transposable elements (TEs) that are polymorphic between the C57BL/6J and the A/J mouse strains were obtained from two different sources. The first one corresponded to a supplementary file from the recent publication of Nellåker *et al.* (2012), in which the authors developed a comprehensive catalog of TE variants across 18 mouse strains (Nellåker *et al.* 2012). The second source corresponded to the MouseIndelDB database (http://variation.osu.edu/mouse_indel/index.html), which reports on structural variants that show polymorphisms between four inbred strains (Akagi *et al.* 2008, 2010). From both databases, we extracted the locations of elements that showed either “insertion” (*i.e.*, present in C57BL/6J but absent in A/J) or “deletion” (*i.e.*, present in A/J but absent in C57BL/6J) *vs.* the mm9 reference sequence of the whole genome from C57BL/6J. For simplicity, the aforementioned publications used the convention of referring to these two types of structural variants as polymorphic “insertion-deletions” (indels). Although the number of polymorphic TEs reported by MouseIndelDB is lower than that reported by Nellåker *et al.* 2012. (Supporting Information, Table S1), sequences in MouseIndelDB have been characterized in greater detail and contain useful annotations. Of note, the vast majority of TE sequences present in a given species are “fixed,” so that in mice, they will be present in both C57BL/6J and A/J and thus nonpolymorphic between the two strains. To obtain data on the abundance of “fixed” TEs in mice, lists of TEs located within all protein-coding genes were downloaded from the TranspoGene database (http://transpogene.tau.ac.il). Recombination rates across the mouse genomes corresponded to the values calculated in a recent report (Brunschwig *et al.* 2012).

Data on genomic binding sites for several factors were obtained from several sources. A list of 33,172 regions associated with CCCTC-binding factor (CTCF) in chromatin from the hearts of adult C57BL/6J mice (as assessed by chromatin immunoprecipitation and massively parallel sequencing) was obtained from the ENCODE/LICR database of transcription factor binding sites using a custom track in the UCSC Genome browser. These data (Release 2, April 2012) correspond to the results of experiments performed by the laboratory of Bing Ren at the Ludwig Institute for Cancer Research. Regions corresponding to binding sites of transcription factors Gata4, Mef2A, Nkx2.5, Srf, and Tbx5 in cardiac chromatin (amounting to either 16,753, 1337, 20,573, 23,806, or 55,582 regions, respectively) corresponded to those published by Schlesinger *et al.* (2011). The 10,486 regions corresponding to the abundance of acetylated histone 3 sites and the 3596 binding sites for the p300 histone acetylase in cardiac chromatin corresponded to those published by Blow *et al.* (2010) and Schueler *et al.* (2012), respectively.

Lists of other eQTLs obtained in RIS were downloaded from the www.genenetwork.org web site using the Genograph tool. In short, the tool uses WebQTL to detect all eQTLs associated to the expression levels of genes within a given dataset (Chesler *et al.* 2005). Datasets used are listed in Table S2. In addition to data for whole eyes from AxB/BxA mice, we used data obtained with five tissues (eye, kidney, hippocampus, hypothalamus, and cerebrum) from BxD mouse RIS. The latter originate from crosses between the parental C57BL/6J (B6) and DBA/2J strains. After analysis, eQTLs were selected using a false-discovery rate threshold of 0.2. As for our own data, we defined *cis*-eQTLs as those whose peak eQTL was within 1 MB of the physical location of the corresponding gene start. Using the same parameters as for detection of *cis*-eQTL clusters (*i.e.*, boxes containing at least three *cis*-eQTL separated by maximum interval of 500 kb), we calculated for each pair of analyzed tissues which proportion of *cis*-eQTL−containing regions overlapped between the two datasets.

### Selection and comparative analysis of genomic regions

Clusters of *cis*-eQTLs were detected by defining regions in which *cis*-eQTLs were separated by maximum distances of either 250, 500, or 750 kb. Control clusters were defined using the same maximum intervals between genes detected by the Illumina array in mouse hearts and imposing a maximal limit on the overall size of control clusters to obtained clusters whose size was not significantly different from that of matching *cis*-eQTL clusters (Table S3). To further verify that both types of clusters had similar properties, we calculated the number of “Entrez” genes in each cluster using the biomaRt R package (version 2.10.0) (Durinck *et al.* 2009) interfaced to BioMart databases. Coexpression levels were quantified by calculating the absolute value of the Pearson correlation coefficient among expression levels of detected genes in the *cis*-eQTL clusters and compared with the coexpression levels observed in two other kinds of boxes: (1) control clusters (whose characteristics were similar to cis eQTL clusters in terms of size, number of genes detected in the heart by the Illumina microarray, total number of genes, and overall level of expression of detected genes); and (2) random regions (corresponding to boxes of similar size chosen randomly within the genome).

For comparisons of the abundance of structural variants and/or binding sites of regulatory factors, the regions analyzed were slightly larger than the clusters themselves and were selected by adding flanking regions of either 250, 500 or 1000 kb to four types of boxes: (1) the same *cis*-eQTL and control clusters defined above (using maximum intervals between *cis*-eQTLS or detected genes of either 250 or 500 kb; and (2) regions with the same size as the previous ones but either centered around single *cis*-eQTLs or selected randomly throughout the genome. Data calculated represented the number of features per Mb in each different region (*cis*-eQTL cluster, control cluster, and random region). For easy comparison across different types of features, all data were normalized by dividing them by the mean number of features (*i.e.*, structural variants or binding sites) found in the random group. Accordingly, the mean normalized number of features in random groups was 1 (± SD), and the values in other regions corresponded to “fold difference” compared to random regions.

Motif searches were performed in the sequences of polymorphic short interspersed elements (SINE) and long-terminal repeat (LTR)-TEs, using the HOMER bioinformatic package (http://biowhat.ucsd.edu/homer/motif). Sequences analyzed corresponded to those that were present in C57BL/6 but absent in A/J (referred to as C57(+)/A/J(−) in Table 1), as these were the only ones where full sequence information was available. To test whether the regions containing polymorphic SINEs had specific characteristics, we use the “annotate_peaks” function provided by the HOMER package.

**Table 1:** Properties of regions containing polymorphic TEs

	LTR C57 (+)	LTR C57 (−)	SINE C57 (+)	SINE C57 (−)
Genomic location				
Intron	10	22	42	42
Intergenic	25	44	20	29
Promoter	1	3	1	2
TSS		1	1	3
3 UTR		1		
Exon				1
Intergenic/intronic detailed annotations				
No specific feature	1	31	1	49
SINE		7	61	
LTR	34	9		12
LINE		9		9
DNA repeat		3		1
Low complexity		1		
Simple repeat		6		

TEs, transposable elements; LTR, long-terminal repeat; SINE, short interspersed element; TSS, transcription start site; UTR, untranslated region; LINE, long interspersed element.

### Statistics

Comparisons between groups were performed by Student’s *t*-test (in the case of 2 groups) or by one-way analysis of variance followed be Tukey’s honestly significant difference post-hoc multiple comparison tests (in the cases of comparisons between more than two groups). Differences in the relative abundance of total and polymorphic TEs in different genomic regions were tested by two-way analysis of variance followed by Tukey’s honestly significant difference post-hoc tests.

## Results

### Detection of *cis*-eQTL clusters

Using the Illumina MouseRef-8 microarray, we detected a total of 8725 genes expressed in hearts from the AXB/BXA mice. Further genomic mapping revealed that 777 of these genes were linked to *cis*-eQTLs, and several of them formed clusters of three or more *cis*-eQTLs within genomic intervals of a few hundred kilobases. We thus tested several window sizes to best define *cis*-eQTL clusters and control clusters (Table S3). By using maximum intervals of 250 kb, we detected a total of 42 *cis*-eQTL clusters (containing in average 4.23 ± 1.9 detected genes, ranging from 3 to 11, within intervals averaging 221.9 ± 130 kb), and 188 control clusters (containing in average 4.75 ± 1.85 detected genes, ranging from 3 to 13, within intervals averaging 248 ± 77.6 kb). By using maximum intervals of 500 kb, we detected a total of 53 *cis*-eQTL clusters (containing in average 4.9 ± 3.5 *cis*-eQTL genes, ranging from 3 to 19, within intervals averaging 467 ± 486 kb), and 59 control clusters (containing in average 5.2 ± 2.5 detected genes, ranging from 3 to 17, within intervals averaging 456 ± 119 kb). There were no significant differences between *cis*-eQTL and control clusters for any of the aforementioned values (Table S3). Because genes detected by the Illumina array in heart extracts do not correspond to all genes present in the genome, we also verified the density of all Entrez-annotated genes in the same regions to estimate total gene density, and found no significant difference between *cis*-eQTL and control clusters. When using maximum intervals of 750 kb, we detected a total of 61 *cis*-eQTL clusters but detected only 21 matching control clusters (Table S3). Further analyses were thus restricted to the “250 kb” and “500 kb” clusters. None of the *cis*-eQTL clusters corresponded to clusters of paralogous genes known to arise from tandem duplication. The coordinates of all 42 “250 kb” *cis*-eQTL clusters are listed in Table S9, along with the symbols of corresponding *cis*-eQTL genes. All control clusters and random regions are listed in Table S10 and Table S11.

Although SNPs within probes are not likely to affect the hybridization efficiency of Illumina microarray probes (Schurmann *et al.* 2012), we nonetheless used data from the Sanger website to detect all high-quality (score > 100) SPs that are polymorphic between A/J and C57Bl/6J and verified that no *cis*-eQTL within the clusters could represent an artifact resulting from a SNP polymorphism. Accordingly, we found a total of 91 SNPs falling within probe sequences. Among them, eight corresponded to *cis*-eQTLs, but none of them corresponded to those found in the *cis*-eQTL clusters.

**Figure 1:**
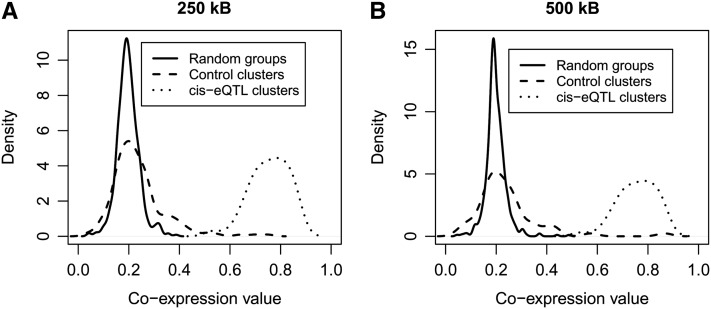
Distribution plots of coexpression values (calculated as Pearson’s coefficients) of genes in *cis*-eQTL clusters (dotted line), control clusters (dashed line), and random boxes (solid line). For each cluster, the coexpression values represent the mean of all pairwise coexpression values between genes in the cluster. The absolute coexpression values (mean ± SD) was 0.76 ± 0.07 in *cis*-eQTL clusters; this value was significantly greater (*P* < 0.001) than that obtained for genes in either control clusters (0.26 ± 0.12) or random groups of genes (0.19 ± 0.19). (A) Coexpression levels in “250 kb” clusters. (B) Coexpression levels in “500 kb” clusters.

Coexpression levels were quantified by calculating the absolute values of the Pearson correlation coefficients between each pair of genes within clusters. Within each *cis*-eQTL cluster, overall coexpression levels were calculated by the averaging all pairwise coexpression values and then compared with values obtained in either corresponding control clusters or in 500 “random groups.” The latter comprised either 3 to 11 genes (for the “250 kb” clusters) or 3 to 19 genes (for the “500 kb” clusters), all genes being chosen randomly throughout the genome. For *cis*-eQTL genes within “250 kb” clusters, coexpression level was 0.755 ± 0.07 (mean ± SD), this value being significantly greater (*P* < 10e-16) than that obtained for detected genes in control clusters (0.23 ± 0.09). Although mean coexpression level in control clusters was about 17% greater than in random groups of genes (0.196 ± 0.04, *P* = 7.4e-07), it was also more than three times lower than that found *in cis*-eQTL clusters (Figure 1A). Very similar results were obtained in terms of coexpression levels and intergroup differences when using the “500” kb clusters (Figure 1B). The increased coexpression of genes within the *cis*-eQTL clusters was not due to an overall greater level of expression of genes in the cluster: the mean log2 value of expression level of genes within the *cis*-eQTL clusters was 9.023, this value being not significantly different (*P* = 0.9) from the level of expression of genes in control clusters (*i.e.*, 9.02).

Given the average size of *cis*-eQTL clusters (*i.e.*, 248 to 456 kb), the average size of intervals between polymorphic SNPs in the RIS panel (2.59 ± 2.95 Mb) and the high level of linkage disequilibrium between adjacent informative SNPs (r^2^ = 0.8), the vast majority of neighboring and coexpressed *cis*-eQTLs within *cis*-eQTL clusters are likely to have the same allelic origin. In contrast, *cis*-eQTL genes within *cis*-eQTL clusters did not show homogeneity either in terms of regulation (because consistent up- or down-regulation of *cis*-eQTL genes was found in only 26% of the *cis*-eQTL clusters) or in terms of genomic strand origin (because both strands of genomic DNA contributed to the sequences of neighboring and co-expressed *cis*-eQTL in 77% of the *cis*-eQTL clusters). There was little evidence to suggest that the *cis*-eQTL clusters could correspond to either recombinant blocks or to regions with different recombination rates. When defining minimal haplotype blocks as regions flanked by polymorphic markers, we found a total of 930 blocks whose average size (2.59 ± 2.95 Mb) was considerably larger than that of *cis*-eQTL clusters (248 to 456 kb). Moreover, the average coexpression value of detected genes within these minimal haplotype blocks was 0.25 ± 0.11 and thus much lower than that of *cis*-eQTLs within *cis*-eQTL clusters (0.755 ± 0.07). Thus, high coexpression levels were found only for *cis*-eQTL genes within *cis*-eQTL clusters, and not for all detected genes throughout the haplotype blocks. Finally, the distribution of recombinant rate values *in cis*-eQTL clusters did not appear to be different from that of detected genes in control clusters (Figure S1).

### Structural characteristics of regions containing *cis*-eQTL clusters

The detection of clusters of *cis*-eQTLs suggested that genetic polymorphisms in some regions could associate with changes in the expression levels of several genes in the same region. Considering that it would be unlikely that such coordinate changes in the regulation of several neighboring genes could result from SNPs each affecting the expression of corresponding *cis*-eQTL genes in a proportionate manner, we mined databases to question whether *cis*-eQTL cluster regions could show enrichment in structural variants (with potential of affecting expression of all *cis*-eQTLs in the region). Because the majority (*i.e.*, 98%) of mouse structural variants have been reported to correspond to TE variants (Akagi *et al.* 2008; Yalcin *et al.* 2011), we used data from the most recent report that established a catalog of TE variants across mouse strains (Nellåker *et al.* 2012) to test whether *cis*-eQTL clusters and their surrounding regions would contain more TE variants than either control clusters or regions of similar size centered around single *cis*-eQTLs. The respective abundances of TEs that were reported as polymorphic between A/J and C57BL/6J are listed in Table S1. Considering that regulatory regions can be located either upstream or downstream of the genes under consideration, we defined the regions to be analyzed by adding flanking sequences with lengths of either 250, 500, or 1000 kb to the regions corresponding to both types of clusters, thus corresponding to regions of six different sizes (Table S4 and Table S5).

**Figure 2:**
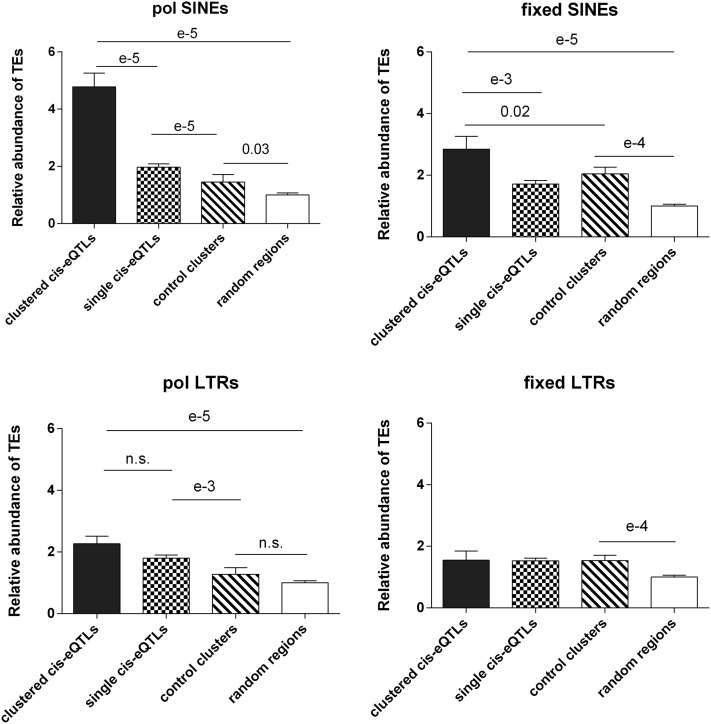
Relative abundance of polymorphic and fixed TEs (SINEs and LTR-TEs) in *cis*-eQTL clusters, control clusters, single *cis*-eQTL regions, and random boxes. Each bar represents mean ± SEM of the relative abundance of TEs in each genomic region box (absolute number of TEs in each box divided by the mean value of the number of TEs in the corresponding random boxes). *P* values are as indicated.

**Figure 3:**
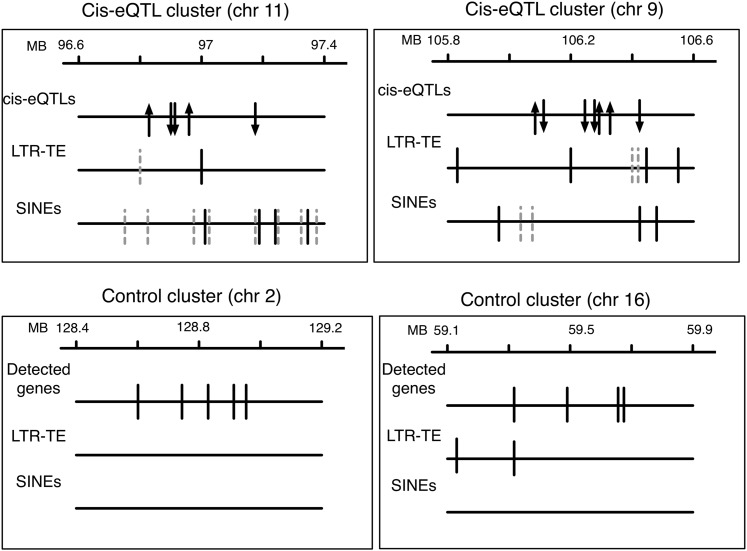
Representative examples illustrating two *cis*-eQTL clusters and control clusters of matching size. For each cluster, the first line (on top) represents respective genomic scales (in Mb); the second line represents the genomic positions of either *cis*-eQTLs or detected genes; and the third and fourth lines represent the genomic positions of polymorphic LTR-TE and SINEs. On the second line, *cis*-eQTL genes are represented at their respective position by arrows; the arrow for the first gene on the left points upwards; other genes are represented by either upwards or downward arrows, according to whether they correlated positively or negatively, respectively, with the change in expression of the first gene. On the third and fourth lines, plain vertical lines correspond to TEs that are C57(+)/A/J(−); dashed lines correspond to TEs that are C57(−)/A/J(+).

For each of the six sizes of regions, comparisons were made between the three types of “defined” regions (*cis*-eQTL clusters, control clusters, and regions centered on single *cis*-eQTLs) and the fourth type of region, consisting of random regions of matching size. Overall, the same interregion differences were found regardless of how the regions were defined. For simplicity, the regions corresponding to the “250 kb” clusters augmented by flanking regions of 250 kb were chosen as representative data for presentation (Figure 2). Long interspersed elements (LINEs) and LINE fragments showed some minor (usually nonsignificant) differences in abundance between the four types of regions. Both polymorphic and fixed SINEs and LTRs were more abundant in all three defined regions than in random regions. For SINEs, this finding is in keeping with the fact that these elements are generally more abundant in gene-rich than in gene-poor regions (Jurka *et al.* 2005). However, for polymorphic TEs, only SINEs were significantly greater (*P* < e-5) *in cis*-eQTL clusters compared with both control clusters and regions centered on single eQTLs. Moreover, the fold-enrichment and the significance of these differences were of greater magnitude for polymorphic SINEs than for fixed SINEs, which indicated that the greater abundance of polymorphic SINEs *in cis*-eQTL clusters was not a mere consequence of total abundance of TEs but rather a consequence of the genetic differences between C57BL/6J and A/J mice. These differences were not due to differences in total gene density because the abundance of total genes *in cis*-eQTL regions (10.1 ± 17) was not significantly different than that in control regions (10.18 ± 3.5). Representative examples that compare two *cis*-eQTL clusters and control clusters of matching sizes are shown in Figure 3. Altogether, polymorphic SINEs appeared to be a signature characteristic of *cis*-eQTL regions. We also found that the density of polymorphic SINEs *in cis*-eQTL cluster regions (taken along with their 250 kb flanking regions) was calculated to be 10.3 ± 10.7 polymorphic SINEs/MB; this value was significantly greater (*P* < 0.002) than that found in other regions of corresponding haplotype blocks outside of the *cis*-eQTL clusters (5.1 ± 8.1). This finding provided additional evidence that the *cis*-eQTL clusters had features that differentiated them from the haplotype blocks that contained them.

To test the possible functional impact of polymorphic SINEs, motif enrichment analyses were performed for TE sequences that are present in C57BL/6 and deleted in A/J mice (because the full sequences of TEs deleted in C57BL/6 and present in A/J are not available yet). The list of most significantly enriched binding sites is shown in Table S6. For polymorphic SINEs, the binding site that was most statistically enriched (*P* = 1e-1283) was that previously reported to be bound by BORIS, a CTCF paralogue that binds a CTCF-like binding site (Pugacheva *et al.* 2010). This site matched the composition of the M1 moiety of the full CTCF binding site recently described by Schmidt *et al.* (2012). Most other significantly enriched sites corresponded to binding sites for transcription factors from several families, in addition to another CTCF binding site that in fact matches the composition of the M2 moiety of the full CTCF binding site (Schmidt *et al.* 2012).

**Figure 4:**
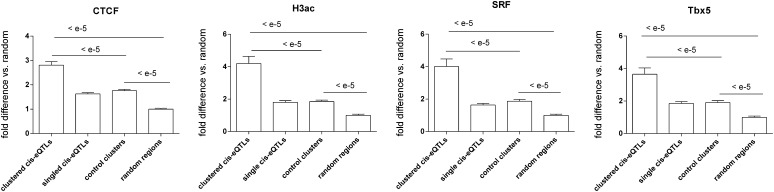
Relative abundance of binding sites for CTCF, SRF, and Tbx5 and of the H3Ac chromatin marks previously reported in chromatin from mouse hearts *cis*-eQTL clusters, control clusters, single *cis*-eQTL regions, and random boxes.

According to MouseIndelDB annotation, 19% of SINEs that are polymorphic between C56BL/6J and A/J correspond to B1 SINEs, with the remainder corresponding to B2 SINEs. We also investigated the nature of regions harboring polymorphic TEs (Table 1). Notwithstanding a few exceptions, the great majority of polymorphic TEs fell into either intronic or intergenic regions. Not surprisingly, C57(+)/A/J(−) LTR-TEs and SINEs fell into C57 regions previously annotated as having these characteristics. Interestingly, C57(−)/A/J(+) LTR-TEs sometimes fell into regions annotated as containing LINEs or SINEs in C57BL/6J, whereas C57(−)/A/J(+) SINEs sometimes fell into regions already containing LTR-TEs or LINEs. Beyond polymorphic TEs, we obtained from previous publications (reporting the results of Chip-Seq experiments performed on mouse heart chromatin) lists of all regions corresponding to either binding sites for transcription factors or chromatin modifications. We then tested whether all corresponding genomic features showed differential abundance between *cis*-eQTL, control, and random regions by using the six different types of window sizes described previously (Table S7 and Table S8). Overall, the same interregion differences were found regardless of how the regions were defined, but differences tended to be more pronounced for regions surrounding and comprising the “250 kb” clusters. Accordingly, the regions corresponding to the “250 kb” clusters augmented by flanking regions of 250 kb were chosen as representative examples for presentation (Figure 4). Overall, the abundance of all binding sites was lower in random regions than in all types of defined regions. For some regulatory factors (CTCF, H3Ac, SRF, and Tbx5), their abundance was much greater *in cis*-eQTL regions than in the other two types of defined regions control regions and more abundant in the latter than in random regions (Figure 4).

### Comparisons with other panels of RIS

To test to which extent *cis*-eQTL clusters would be conserved across tissues, we questioned whether regions containing *cis*-eQTL clusters for cardiac genes would overlap with regions containing *cis*-eQTL clusters for genes expressed in another tissues (with clusters of *cis*-eQTLs being defined on the basis of maximum intervals of 250 Mb between each *cis*-eQTL). We first analyzed gene expression data obtained in AxB/BxA eyes with Illumina microarrays (Table S2), which allowed us to detect a total of 35 *cis*-eQTL clusters (while verifying, as explained previously, that none of the *cis*-eQTL genes could represent an artifact due to the presence of a SNP polymorphism within the sequence of the probes). Despite the fact that the latter data were obtained by other investigators, 12 of the 42 regions containing *cis*-eQTLs clusters for genes expressed in AxB/BxA hearts also contained *cis*-eQTL clusters for genes expressed in eyes from the same RIS.We also analyzed gene expression data for other tissues from the BxD RIS mouse panel, where gene expression was analyzed (Table S2) with either the Affymetrix MoGene 1.0 ST microarray (for hypothalamus) or with the Affymetrix Mouse Genome 430 microarray (for eye, kidney, hippocampus, and cerebellum). Of note, consultation of the list of SNP polymorphisms between C57BL/6J and DBA/2 mice revealed a total of 0.005% and 0.0006% of probes used by the Affymetrix MoGene 1.0 ST and Affymetrix Mouse Genome 430 microarrays, respectively, are affected by such polymorphisms. Corresponding genes were excluded from the *cis*-eQTL analysis. Analysis of the gene expression data from BxD RIS tissues allowed us to detect 52, 279, 260, 77, and 36 *cis*-eQTL clusters for kidneys, eyes, hippocampus, hypothalamus, and cerebrum, respectively. Again, high proportions of *cis*-eQTL−containing regions were shared across tissues. For instance, of the 52 regions detected for genes expressed in kidneys, the number of regions also detected for genes from other tissues amounted to 44 for eyes, 44 for hippocampus, 15 for hypothalamus, and 15 for cerebrum. Finally, to test whether regions containing *cis*-eQTL clusters in one tissue would overlap with regions containing *cis*-eQTL clusters in one same tissue from two RIS panels, we compared data obtained for eye genes from AxB/BxA and BxD RIS. Of the 35 regions containing *cis*-eQTL clusters for genes from AxB/BxA eyes, 16 corresponded to regions containing *cis*-eQTL clusters for genes from BxD eyes.

## Discussion

Despite evidence suggesting the existence of gene coexpression domains in several eukaryotic organisms (Michalak 2008; Elizondo *et al.* 2009), data available to date suggested that coexpression in mammals rarely concerns more than doublets or triplets of immediately adjacent genes (Sémon and Duret 2006; Purmann *et al.* 2007). Our data show that within panels of mouse RIS, where gene expression variability is due in part to genetic polymorphisms, it is indeed possible to detect short-range clustering of more than three neighboring (but not necessarily adjacent) coexpressed genes. Moreover, all coexpressed genes within these domains showed linkage to loci having the same position as that of the domains, thus showing that genetic polymorphisms can associate with the expression levels of several neighboring genes within these domains. Depending on the sizes of windows used to define them, *cis*-eQTL clusters detected for AxB/BxA hearts contained in average 4.2 to 4.9 *cis*-eQTL genes within intervals averaging 248-456 kb. The number of *cis*-eQTL clusters detected for hearts from AxB/BxA panel (*i.e.*, 53) was within the same range as the numbers of *cis*-eQTL clusters detected with three other tissues from the distinct BxD RIS panel (*i.e.*, 60 for cerebrum, 74 for kidneys, and 96 for hypothalamus). Moreover, a great proportion of the regions containing *cis*-eQTL for AxB/BxA hearts were identical to those containing *cis*-eQTL clusters for eyes from either AxB/BxA or BxD RIS, and many of the regions containing *cis*-eQTL clusters for BxD kidneys overlapped with those detected for four other tissues from the same panel. Altogether, the data indicate that *cis*-eQTL clusters occur in a fairly robust and consistent manner across different tissues and/or genetic backgrounds in mouse RIS. Of note, all tissues do not necessarily express the same genes, which may explain in part why some *cis*-eQTL clusters are distinct between tissues. With some tissues (eyes and hippocampus from BxD RIS), up to 300 *cis*-eQTL clusters were detected. A comprehensive list of all genomic regions having potential to contain *cis*-eQTLs and a full understanding of the extent to which they overlap would require data from a more exhaustive list of tissues.

One distinct feature of *cis*-eQTL clusters was that *cis*-eQTL genes in these regions showed a level of coexpression that greatly exceeded that found for detected genes in control regions with similar gene density. For instance, in regions selected as controls for the “500 kb” *cis*-eQTL clusters, only 2 of 59 regions displayed coexpression levels greater than 0.56 (*i.e.*, the value corresponding to the lowest value for gene coexpression *in cis*-eQTL clusters). The *cis*-eQTL clusters thus corresponded to “gene coexpression domain” QTLs. The fact that all coexpressed genes within domains showed linkage to a common locus suggested that the genome contains polymorphisms that can alter coexpression levels. Reasoning that identification of the nature of such polymorphisms could reveal insights as to what drives coexpression of genes within coexpression domains, we mined databases to test whether the *cis*-eQTL regions harbored any particular types of polymorphic structural variants. Accordingly, we found that polymorphic SINEs [either C57(+)/A/J(−) or C57(−)/A/J(+)] were significantly more abundant *in cis*-eQTL clusters than in any other type of control regions. Given the possibility that polymorphic SINEs could in fact drive these high levels of coexpression in corresponding domains, we tested whether they showed enrichment for particular motifs. In addition to binding sites for various transcription factors belonging to different families, polymorphic SINEs showed enrichment for two sites corresponding to CTCF-binding regions. This finding is an agreement with the previous report in which the authors showed that CTCF-binding regions in the mouse genome were preferentially embedded in B2 SINE elements (Bourque *et al.* 2008). B2 SINEs constitute types of SINEs that are specific to rodent genomes, where they have undergone waves of amplification (Kass *et al.* 1997). Accordingly, 81% of the SINEs that are polymorphic between C57BL/6J and A/J were in fact B2 SINEs. Moreover, we compared the relative abundance of either chromatin marks or binding sites for either CTCF or cardiac transcription factors *in cis*-eQTL clusters *vs.* all three other types of regions. Accordingly, we found that binding sites for the transcription factors SRF and TBX5, the chromatin-organizing factors CTCF and p300, and the H3Ac chromatin were all significantly enriched *in cis*-eQTL clusters *vs.* all three other regions.

SINEs and CTCF binding sites are of particular interest. The structural and regulatory organization of the mammalian genome is fundamentally dependent on CTCF, which has been dubbed the “master weaver of the genome” (Phillips and Corces 2009). CTCF generally acts as an insulator preventing the spread of inactive heterochromatin, and is often associated with open chromatin (The ENCODE Project Consortium 2012). This may be particularly pertinent in the context of our current data further documenting the existence of genetically controlled gene coexpression domains. As a matter of fact, recent genome-wide studies on chromatin structure have revealed that mammalian genomes are organized into topological domains, the boundaries of which show enrichment for SINEs and CTCF binding, and where the spread of heterochromatin is constrained (Dixon *et al.* 2012). Likewise, it was recently shown that in several mammalian species, CTCF-binding events are associated with waves of retrotransposon expansion, thus revealing the mechanism by which there are born (Schmidt *et al.* 2012). Our data extend these previous reports in several ways: (1) in addition to interspecies differences, polymorphic SINEs can cause differential abundance of CTCF binding sites between strains of one same species; (2) the CTCF-dependent organization of genomic mammalian domains may not be static because polymorphic SINEs could reshape that organization; and (3) polymorphic SINEs and CTCF-binding sites may constitute a mechanism defining gene coexpression domains, for which there was so far little evidence in mammals beyond doublets of triplets of genes.

Of note, the allelic origin of the *cis*-eQTL cluster regions did not affect the expression of all corresponding *cis*-eQTL in a consistent manner (as illustrated in Figure 3). However, because *cis*-eQTL clusters contained an average of five polymorphic TEs, changes in corresponding chromatin domains may be more complex than simply corresponding to a chromatin structure that is entirely open or closed for the whole region. Moreover, TEs can affect single gene expression by a variety of different possible mechanisms, as for instance by providing alternative promoters or enhancers, serving as insulators or transcriptional silencers, disrupting the exon-intron structure and/or causing premature transcriptional termination (Gogvadze and Buzdin 2009). TE might also affect gene regulation by mechanisms other than just providing regulatory elements. For instance, we found that some C57(−)/A/J(+) polymorphisms fell themselves within regions containing other TEs, and might thus disrupt in A/J the organization of some TE elements that have regulatory effects within the C57BL/6J strain. In combination with complex changes in chromatin structure, all the above mechanisms might account for the nonuniform effects of the allelic origin of *cis*-eQTL clusters on gene expression.

Close to half of mammalian genomes is derived from ancient TEs (primarily retroelements) (van de Lagemaat *et al.* 2003). Given their great abundance in the genome and the emerging recognition of their role in gene regulation, TEs that show polymorphism between inbred strains are increasingly being recognized as potential players in the genetics of quantitative traits (Nellåker *et al.* 2012). Accordingly, some classes of polymorphic TEs have been reported to show small but significant enrichment in refined genomic intervals selected on the basis of previous detection of quantitative trait loci for mouse quantitative traits (Nellåker *et al.* 2012). In terms of gene regulation, recent reports on the effects of TEs have so far concerned mostly single genes (Chernova *et al.* 2008; Palmer and Dulawa 2010; Li *et al.* 2012). However, complex quantitative traits are usually considered to result from the combined regulatory effects of several genes rather than from the highly penetrant effect of single mutations (Manolio *et al.* 2009). Gene coexpression domains might thus be of particular interest, as coordinate dysregulation of the expression levels of several genes within *cis*-eQTL clusters could possibly have greater effects on the phenotypic expression of complex traits than dysregulation of single genes.

Regulatory sequences located outside of coding regions are also interesting in the light of evidence that: (1) up to one third of noncoding sequence variation contributes causally to the traits under investigation in genome-wide association studies (Visel *et al.* 2009); (2) a great proportion of regulatory variants of gene expression are found at a fairly great distance from transcription start sites (Teng *et al.* 2011); and (3) chromatin structure plays important roles in the organization and regulation of our genes (The ENCODE Project Consortium 2012). Genome-wide approaches allowing the discovery and functional characterization of such elements might improve our understanding of their role in human biology and disease susceptibility (Teng *et al.* 2011). However, if polymorphic TEs turn out to have important consequences on gene regulation, appropriate technologies to detect them genome-wide will need to be developed because technologies based on detection of SNP polymorphisms are not sufficient in this regard.

## Supplementary Material

Supporting Information
