# Supplementary material for: Genome-Wide Detection of Gene Coexpression Domains Showing Linkage to Regions Enriched with Polymorphic Retrotransposons in Recombinant Inbred Mouse Strains
Source: G3 (Bethesda). 2013 Apr 1;3(4):597–605. doi: 10.1534/g3.113.005546 (PMC3618347; doi:10.1534/g3.113.005546)
Supplement: Supporting Information [file supp_3_4_597__index.html]

Genome-Wide Detection of Gene Coexpression Domains Showing Linkage to Regions Enriched with Polymorphic Retrotransposons in Recombinant Inbred Mouse Strains — Supporting Information 

# Genome-Wide Detection of Gene Coexpression Domains Showing Linkage to Regions Enriched with Polymorphic Retrotransposons in Recombinant Inbred Mouse Strains

## Supporting Information for Scott-Boyer and Deschepper, 2013

**Files in this Data Supplement:**

- Supporting Information - Figure S1 and Tables S1-S11 (PDF, 284 KB)
- Figure S1 - Distribution of recombination rates in regions corresponding to either cis-eQTL or control clusters (PDF, 77 KB)
- Table S1 - Distribution of recombination rates in regions corresponding to either cis-eQTL or control clusters (PDF, 61 KB)
- Table S2 - Summary of gene expression datasets from mouse RIS (PDF, 59 KB)
- Table S3 - Properties of cis-eQTL and control clusters (defined using three different window sizes) (PDF, 61 KB)
- Table S4 - Normalized abundance of polymorphic and fixed TEs in several sizes of genomic regions around "250 kB" clusters (PDF, 79 KB)
- Table S5 - Normalized abundance of polymorphic and fixed TEs in several sizes of genomic regions around "500 kB" clusters (PDF, 101 KB)
- Table S6 - Most significantly enriched binding sites in polymorphic SINEs (PDF, 65 KB)
- Table S7 - Comparisons for respective normalized abundance of binding sites for regulatory factors in several sizes of genomic regions around "250 kB" clusters (PDF, 84 KB)
- Table S8 - Comparisons for respective normalized abundance of binding sites for regulatory factors in several sizes of genomic regions around "500 kB" clusters (PDF, 106 KB)
- Table S9 - Descriptive information concerning the 42 "250 kB" cis-eQTL clusters (PDF, 90 KB)
- Table S10 - Descriptive information concerning "control clusters" (.xlsx, 19 KB)
- Table S11 - Descriptive information concerning "random boxes" (.xlsx, 23 KB)
