# Supplementary material for: Genome-Wide Detection of Gene Coexpression Domains Showing Linkage to Regions Enriched with Polymorphic Retrotransposons in Recombinant Inbred Mouse Strains
Source: G3 (Bethesda). 2013 Apr 1;3(4):597–605. doi: 10.1534/g3.113.005546 (PMC3618347; doi:10.1534/g3.113.005546)
Supplement: Supporting Information [file supp_g3.113.005546_005546SI.pdf]

G3·Genes | Genomes | Genetics

Genome-wide detection of gene co-expression domains showing linkage to regions enriched with polymorphic retrotransposons in recombinant inbred mouse strains

Marie-Pier Scott-Boyer and Christian F Deschepper[1]

Cardiovascular Biology Research Unit, Institut de recherches cliniques de Montréal (IRCM) and Université de Montréal, Montréal, Québec, H2W 1R7, Canada.

[1]Corresponding author:
Christian F. Deschepper
IRCM
110, avenue des Pins Ouest
Montréal (QC) Canada H2W 1R7
Phone: (514) 987 5759
Fax: (514) 987 5585
E-mail: deschec@ircm.qc.ca

DOI: 10.1534/g3.113.005546

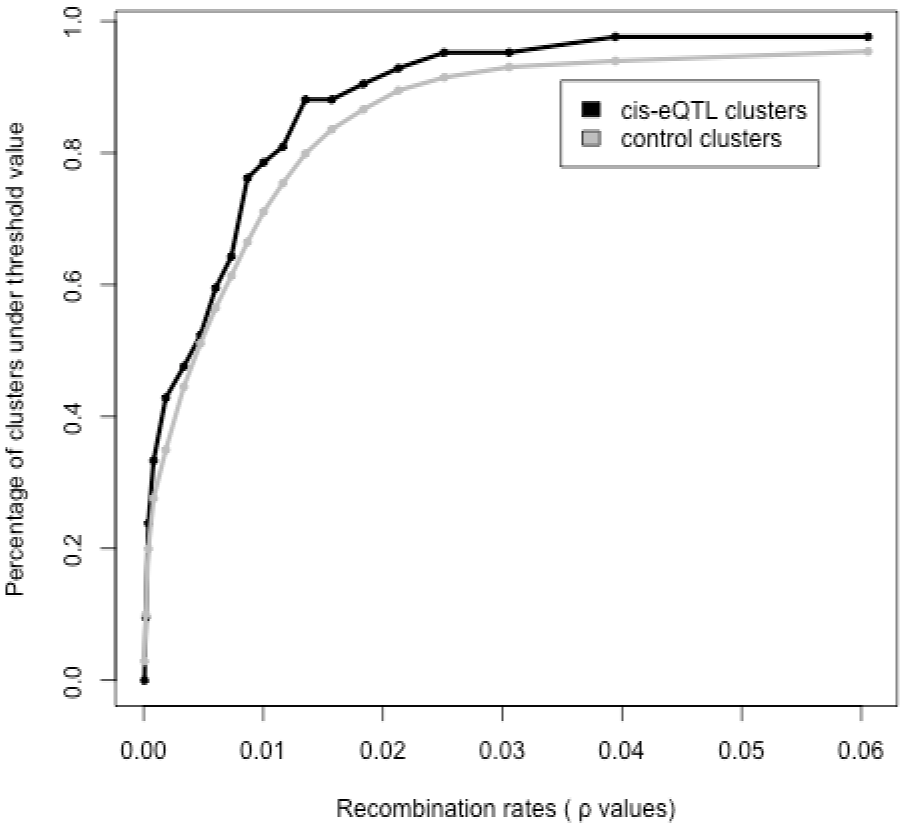

Figure S1 Distribution of recombination rates in regions corresponding to either cis-eQTL or control clusters. The recombination rate values corresponded to the ρ values, as calculated and reported by Brunschwig H et al. (Genetics 191: 757–764, 2012). According to the Chi-square test, there was no significant difference in the distributions of recombination rates in the 2 types of regions ($P > 0.3$).

Table S1 Abundance of polymorphic and total TEs in mouse genomes. Lists of polymorphic TEs were obtained from either the publication of Nellåker et al (2012) or from the MouseIndelDB database. Lists of fixed TEs were obtained from the Transpogene database. n/a: non available.

**Database**	**Genetic origin**	**full LINEs**	**LINE frag.**	**LTR-TEs**	**SINEs**
**Nellåker et al. (polymorphic TEs)**	Both strains	1808	2969	4734	4303
	C57(+) / A/J(-)	606	1015	1901	2378
	C57(-) / A/J(+)	1202	1954	2833	1925
**MouseIndelDB (polymorphic TEs)**	Both strains			2413	1512
	C57(+) / A/J(-)			1436	1512
	C57(-) / A/J(+)			977	0
**Transpogene (fixed TEs)**		78,002	n/a	84,724	190,057

Table S2 Summary of gene expression datasets from mouse RIS. Each line provides information about which tissue and which RIS panel was used for each gene expression dataset, as well as about the number of strains that were profiled and the nature of the microarray platform. The GN access numbers corresponds to the identification number of corresponding datasets in GeneNetwork.

Tissue	RIS panel	# of strains used	Microarray platform	GN access #
Eye	AxB/BxA	26	Illumina MouseRef-6	GN210
Eye	BxD	68	Affymetrix Mouse Genome 430	GN207
Kidney	BxD	54	Affymetrix Mouse Genome 430	GN240
Hippocampus	BxD	67	Affymetrix Mouse Genome 430	GN112
Hypothalamus	BxD	33	Affymetrix MoGene 1.0 ST	GN281
Cerebellum	BxD	28	Affymetrix Mouse Genome 430	GN72

Table S3 Properties of cis-eQTL and control clusters (defined using three different window sizes). In addition to the sizes of the intervals between detected genes, some additional criteria were used in the selection of control clusters in order to have them match cis-eQTL clusters for size and density of total genes. For the 250 kB intervals, only clusters <400 kB and containing more than 3 genes were kept; for the 500 kB, only clusters <650 kB and containing at least 6 genes were kept; for the 750 kB intervals, only clusters of <800 kb and containing at least 8 genes were kept.

**Maximum interval between cis-eQTLs or detected genes**	**250kB**			**500kB**			**750kB**		
	Cis-eQTL clusters	Control cluters	p-val	Cis-eQTL clusters	Control cluters	p-val	Cis-eQTL clusters	Control cluters	p-val
Size of boxes (in kb)	221.9 ±130	248±	0.2	467.1 ±486	456±119	0.87	658.8 ±553	561±123	0.2
Number of detected genes	4.2 ±1.9	4.75±	0.1	4.9 ±3.5	5.23±2.46	0.63	5.1 ±3.6	6.6±3.89	0.11
Total number of genes	7.2 ±6.6	6.30 ±2.9	0.38	12.3 ±16	10.1±3.45	0.35	15.70±17.6	14.9±11.1	0.81
Number of clusters	42	188		53	59		61	21	

Table S4 Normalized abundance of polymorphic and fixed TEs in several sizes of genomic regions around "250 kB" clusters. Normalized abundance of TEs was calculated by dividing the abundance of each element with that found in random regions. Test regions corresponded to those containing cis-eQTLs (either clustered or single) or control clusters, each region being augmented by flanking regions of either 250, 500, 1000 kb.

		Polymorphic				Fixed	
		**full LINEs**	**LINE frag.**	**LTR-TEs**	**SINEs**	**LTR-TEs**	**SINEs**
**Size of flanking regions**	250 kB						
**Normalized abundance of TEs / MB (mean ± SD)**	Cis-eqtl clusters (n=42)	0.51 ± 1.39	0.77 ± 1.42	2.27 ± 1.75	4.78 ± 3.51	1.55 ± 2.17	2.85 ± 3.01
	CTL clusters (n=188)	0.46 ± 1.16	0.55 ± 1.01	1.28 ± 1.58	1.45 ± 2.07	1.53 ± 1.3	2.05 ± 1.62
	Single cis-eQTL regions (n=232)	0.59 ± 1.32	0.94 ± 1.33	1.8± 1.53	1.97±1.84	1.53±1.26	1.71±1.78
	Random regions (n=500)	1 ± 2.04	1 ± 1.72	1 ± 1.39	1 ± 1.72	1 ± 1.27	1 ± 1.42
**Anova P values**		0.00039	0.0059	1.22E-13	1.11E-33	3.15E-08	4.40E-20
**Post-hoc Tukey P values**	Cis-eqtl clusters vs CTL	0.99	0.83	0.00053	<e-5	0.99	0.02306
	Cis-eqtl clusters vs single cis-eQTL regions	0.99	0.90	0.22	<e-5	0.99	0.00023
	Cis-eqtl clusters vs random	0.28	0.77	<e-5	<e-5	0.048	<e-5
	Control and single cis-eQTL regions	0.8	0.042	0.0019	0.029	0.99	0.15
	CTL vs random	0.001	0.003	0.12	0.03	2.00E-05	<e-5
	Random vs single cis-eQTL regions	0.015	0.96	<e-5	<e-5	<e-5	<e-5

		Polymorphic				Fixed	
		**full LINEs**	**LINE frag.**	**LTR-TEs**	**SINEs**	**LTR-TEs**	**SINEs**
**Size of flanking regions**	500 kB						
**Normalized abundance of TEs / MB (mean ± SD)**	Cis-eqtl clusters (n=42)	0.54 ± 1.42	0.98 ± 1.69	1.88 ± 1.43	4.14 ± 2.84	1.39 ± 1.22	2.46 ± 1.86
	CTL clusters (n=188)	0.61 ± 1.16	0.65 ± 1.05	1.10 ± 1.25	1.27 ± 1.59	1.41 ± 1.10	1.79 ± 1.39
	Single cis-eQTL regions (n=232)	0.75±1.24	0.93±1.07	1.62±1.21	1.7±1.45	1.39±1.1	1.53±1.48
	Random regions (n=500)	1 ± 1.77	1 ± 1.51	1 ± 1.20	1 ± 1.6	1 ± 1.10	1 ± 1.29
**Anova P values**		0.0068	0.024	1.12E-11	2.22E-31	1.01E-06	6.32E-17
**Post-hoc Tukey P values**	Cis-eqtl clusters vs CTL	0.99	0.47	0.001	<e-5	0.99	0.024
	Cis-eqtl clusters vs single cis-eQTL regions	0.83	0.99	0.58	<e-5	1	0.00041
	Cis-eqtl clusters vs random	0.23	0.99	5.00E-05	<e-5	0.12352	<e-5
	Control and single cis-eQTL regions	0.75	0.13	8.00E-05	0.03	0.99	0.22
	CTL vs random	0.014	0.013	0.79	0.21	1.00E-04	<e-5
	Random vs single cis-eQTL regions	0.18	0.92	<e-5	<e-5	6.00E-05	1.00E-05

		Polymorphic				Fixed	
		full LINEs	LINE frag.	LTR-TEs	SINEs	LTR-TEs	SINEs
**Size of flanking regions**	1 MB						
**Normalized abundance of TEs / MB (mean ± SD**	Cis-eqtl clusters (n=42)	0.59 ± 1.25	0.93 ± 1.27	1.71 ± 1.17	3.47 ± 2.36	1.26 ± 0.93	2.12 ± 1.54
	CTL clusters (n=188)	0.76 ± 1.26	0.72 ± 1.02	1.06 ± 0.92	1.14 ± 1.17	1.33 ± 0.99	1.53 ± 1.1
	Single cis-eQTL regions (n=232)	0.48±1.71	0.81±1.91	1.76±2.28	2.69±3.3	1.7±1.58	2.02±2.02
	Random regions (n=500)	1 ± 1.47	1 ± 1.39	1 ± 1.03	1 ± 1.39	1 ± 0.87	1 ± 1.11
**Anova P values**		0.00013	0.1	4.95E-11	7.17E-31	1.75E-13	3.84E-20
**Post-hoc Tukey P values**	Cis-eqtl clusters vs CTL	0.91	0.82	0.037	<e-5	0.98	0.069
	Cis-eqtl clusters vs single cis-eQTL regions	0.96	0.96	0.99	0.1	0.083	0.97
	Cis-eqtl clusters vs random	0.32	0.99	0.011	<e-5	0.46	1.00E-05
	Control and single cis-eQTL regions	0.22	0.91	<e-5	<e-5	0.0034	0.0024
	CTL vs random	0.22	0.11	0.96	0.86	0.0034	6.00E-05
	Random vs single cis-eQTL regions	7.00E-05	0.37	<e-5	<e-5	<e-5	<e-5

Table S5 Normalized abundance of polymorphic and fixed TEs in several sizes of genomic regions around "500 kB" clusters. Normalized abundance of TEs was calculated by dividing the abundance of each element with that found in random regions. Test regions corresponded to those containing cis-eQTLs (either clustered or single) or control clusters, each region being augmented by flanking regions of either 250, 500, 1000 kb.

		Polymorphic				Total	
		**full LINEs**	**LINE frag.**	**LTR-TEs**	**SINEs**	**LTR-TEs**	**SINEs**
**Size of flanking regions**	250 kB						
**Mean numbers of elements /MB (fold diff vs random mean)**	Cis-eqtl clusters (n=53)	0.66 ± 1.33	0.96 ± 1.59	2.24 ± 1.73	4.00 ± 2.84	1.57 ± 1.99	2.85 ± 2.8
	CTL clusters (n=59)	0.89 ±1.71	0.72 ± 0.9	1.08 ± 1.1	1.29 ± 1.48	1.36 ± 1.17	1.91 ± 1.43
	Single cis-eQTL regions (n=232)	0.69±1.26	0.97±1.19	1.69±1.29	1.76±1.6	1.43±1.14	1.64±1.6
	Random regions (n=500)	1 ± 1.9	1 ± 1.56	1 ± 1.28	1 ± 1.6	1 ± 1.19	1 ± 1.33
**Anova P values**		0.099	0.56	8.05E-16	3.05E-32	9.33E-06	1.16E-18
**Post-hoc Tukey P values**	Cis-eqtl clusters vs CTL	0.88	0.8	2.00E-05	<e-5	0.81	0.0078
	Cis-eqtl clusters vs single cis-eQTL regions	0.99	1	0.028	<e-5	0.88	<e-5
	Cis-eqtl clusters vs random	0.5	0.99	<e-5	<e-5	0.008	<e-5
	Control and single cis-eQTL regions	0.84	0.63	0.0071	0.22	0.98	0.62
	CTL vs random	0.96	0.48	0.97	0.59	0.14	0.00012
	Random vs single cis-eQTL regions	0.09	0.99	<e-5	<e-5	8.00E-05	<e-5

		Polymorphic				Total	
		**full LINEs**	**LINE frag.**	**LTR-TEs**	**SINEs**	**LTR-TEs**	**SINEs**
**Size of flanking regions**	500 kB						
**Mean numbers of elements /MB (fold diff vs random mean)**	Cis-eqtl clusters (n=53)	0.69 ± 1.38	1.08 ± 1.73	1.80 ± 1.32	3.41 ± 2.19	1.36 ± 1.16	2.39 ± 1.77
	CTL clusters (n=59)	1.10 ± 1.9	0.88 ± 1.05	1.08 ± 1.07	1.14 ± 1.16	1.32 ± 1.1	1.60 ± 1.28
	Single cis-eQTL regions (n=232)	0.8±1.2	1±1.11	1.56±1.1	1.58±1.25	1.36±1.06	1.49±1.41
	Random regions (n=500)	1 ± 1.67	1 ± 1.49	1 ± 1.15	1 ± 1.54	1 ± 1.03	1 ± 1.22
**Anova P values**		0.19	0.8	2.65E-11	5.09E-27	3.93E-05	2.80E-14
**Post-hoc Tukey P values**	Cis-eqtl clusters vs CTL	0.49	0.86	0.0046	<e-5	0.99	0.0092
	Cis-eqtl clusters vs single cis-eQTL regions	0.96	0.97	0.51	<e-5	0.99	5.00E-05
	Cis-eqtl clusters vs random	0.50	0.97	1.00E-05	<e-5	0.088	<e-5
	Control and single cis-eQTL regions	0.52	0.93	0.019	0.17	0.99	0.93
	CTL vs random	0.96	0.91	0.96	0.9	0.117	0.0054
	Random vs single cis-eQTL regions	0.35	0.99	<e-5	1.00E-05	9.00E-05	2.00E-05

		Polymorphic				Total	
		**full LINEs**	**LINE frag.**	**LTR-TEs**	**SINEs**	**LTR-TEs**	**SINEs**
**Size of flanking regions**	1 MB						
**Mean numbers of elements /MB (fold diff vs random mean)**	Cis-eqtl clusters (n=53)	0.76 ± 1.21	1.06 ± 1.34	1.58 ± 1.04	2.93 ± 1.87	1.18 ± 0.86	2.02 ± 1.45
	CTL clusters (n=59)	1.01 ± 1.43	0.94 ± 1.08	1.03 ± 0.91	1.06 ± 1.02	1.16 ± 0.85	1.31 ± 0.95
	Single cis-eQTL regions (n=232)						
	Random regions (n=500)	1 ± 1.4	1 ± 1.36	1 ± 0.99	1 ± 1.35	1 ± 0.84	1 ± 1.07
**Anova P values**		1.97E-05	0.42	8.00E-10	3.84E-26	3.43E-13	4.94E-19
**Post-hoc Tukey P values**	Cis-eqtl clusters vs CTL	0.78	0.97	0.16	1.00E-05	0.9	0.04
	Cis-eqtl clusters vs single cis-eQTL regions	0.47	0.7	0.9	0.75	0.012	0.99
	Cis-eqtl clusters vs random	0.65	0.99	0.022	<e-5	0.65	<e-5
	Control and single cis-eQTL regions	0.034	0.9	0.004	0	0.0047	0.0036
	CTL vs random	0.99	0.99	0.99	0.99	0.72	0.39
	Random vs single cis-eQTL regions	1.00E-05	0.39	<e-5	<e-5	<e-5	<e-5

Table S6 Most significantly enriched binding sites in polymorphic SINEs

Motif Name (family)	CHIP-Seq cell line	Consensus	P-value	% of Target Sequences with Motif	% of Background Sequences with Motif
**CTCFL /BORIS (Zinc finger)**	K562	CNNBRGCGCCCCCTGSTGGC	1e-1283	63.96%	3.33%
ZNF143 (Zinc finger)	CUTTL	ATTTCCCAGVAKSCY	1e-1273	73.63%	5.91%
c-Myc	mES	VVCCACGTGG	1e-1213	75.98%	7.34%
n-Myc (Helix-Loop-Helix)	mES	VRCCACGTGG	1e-1116	76.39%	8.77%
Max (Helix-Loop-Helix))	K562	RCCACGTGGYYN	1e-1094	77.54%	9.57%
RUNX (Runt)	HPC7	SAAACCACAG	1e-1000	76.44%	10.61%
Gfi1b (Zinc finger)	HPC7	MAATCACTGC	1e-996	72.28%	8.82%
RUNX2 (Runt)	PCa	NWAACCACADNN	1e-906	76.44%	12.37%
RUNX1 (Runt)	Jurkat	AAACCACARM	1e-820	76.70%	14.39%
Cdx2 (Homeobox)	mES	GYMATAAAAH	1e-623	63.70%	11.95%
Olig2 (Helix-Loop-Helix))	Neuron	RCCATMTGTT	1e-613	79.10%	22.14%
HNF4a (Nuclear Receptors)	HpeG2	CARRGKBCAAAGTYCA	1e-359	33.54%	4.58%
GATA3 (Zinc finger)	iTreg	AGATSTNDNNDSAGATAASN	1e-322	23.19%	1.97%
Erra(NR)	HepG2	CAAAGGTCAG	1.00E-304	62.35%	22.53%
FOXA1 (Forkhead)	MCF7	WAAGTAAACA	1.00E-304	53.46%	16.26%
Smad3 (MAD)	NPC	TWGTCTGV	1.00E-265	68.28%	29.69%
FOXA1 (Forkhead)	LNCaP	WAAGTAAACA	1.00E-265	54.08%	18.54%
AR-halfsite (Nuclear Receptors)	LNCaP	CCAGGAACAG	1.00E-232	78.00%	41.54%
Nkx2.5 (Homeobox)	HL1	RRSCACTYAA	1.00E-182	56.89%	25.78%
Nanog (Homeobox)	mES	RGCCATTAAC	1.00E-106	69.53%	44.63%
NeuroD1 (Helix-Loop-Helix)	Islet	GCCATCTGTT	1.00E-102	25.07%	8.50%
**CTCF (Zinc finger)**	CD4+	AYAGTGCCMYCTRGTGGCCA	1.00E-67	9.26%	1.82%

Table S7 Comparisons for respective normalized abundance of binding sites for regulatory factors in several sizes of genomic regions around "250 kB" clusters. Normalized abundances of binding sites for regulatory factors was calculated in regions containing cis-eQTL cluster, control cluster, single cis-eQTL region or random regions, and augmented by flanking region of either 250, 500, 1000 kb.

		CTSF	h3ac	gata4	MEF2A	NKX2_5	SRF	TBX5	P300
**Size of flanking regions**	250 kB								
**Normalized abundance of TFs / MB (mean ± SD)**	Cis-eqtl clusters (n=42)	2.81 ± 0.94	4.20 ± 2.89	2.10 ± 1.65	2.67 ± 4.8	2.11 ± 1.65	4.02 ± 2.92	3.65 ± 2.52	2.43 ± 2.22
	CTL clusters (n=188)	1.76 ±0.69	1.85 ± 1.19	1.72 ± 1.29	1.47 ± 3.27	1.86 ± 1.62	1.87 ± 1.51	1.91 ± 1.67	1.64 ± 1.8
	Single cis-eQTL regions (n=232)	1.63±0.73	1.81±1.6	1.68±1.4	1.34±2.53	1.64±1.41	1.9±1.85	1.85±1.73	1.7±1.89
	Random regions (n=500)	1 ± 0.89	1 ± 1.57	1 ± 1.46	1 ± 4.16	1 ± 1.63	1 ± 1.58	1 ± 1.57	1 ± 1.61
**Anova P values**		4.4E-56	7.65E-37	6.20E-14	0.026	8.29E-13	5.89E-31	3.68E-27	1.48E-10
**Post-hoc Tukey P values**	Cis-eqtl clusters vs CTL	<e-5	<e-5	0.40684	0.22	0.77	<e-5	<e-5	0.039
	Cis-eqtl clusters vs single cis-eQTL regions	<e-5	<e-5	0.29376	0.140	0.27	<e-5	<e-5	0.059
	Cis-eqtl clusters vs random	<e-5	<e-5	1.00E-05	0.025	7.00E-05	<e-5	<e-5	<e-5
	Control and single cis-eQTL regions	0.41	0.99	0.98	0.98	0.49	0.99	0.97	0.98
	CTL vs random	<e-5	<e-5	<e-5	0.44	<e-5	<e-5	<e-5	0.00014
	Random vs single cis-eQTL regions	<e-5	<e-5	<e-5	0.65	<e-5	<e-5	<e-5	<e-5
	Cis-eqtl clusters vs CTL	<e-5	<e-5	0.4	0.22	0.77	<e-5	<e-5	0.039

		CTSF	h3ac	gata4	MEF2A	NKX2_5	SRF	TBX5	P300
**Size of flanking regions**	500 kB								
**Normalized abundance of TFs / MB (mean ± SD)**	Cis-eqtl clusters (n=42)	2.43 ± 0.78	3.60 ± 2.36	2.06 ± 1.5	2.67 ± 4.69	2.06 ± 1.69	3.47 ± 2.58	3.24 ± 2.17	2.03 ± 1.64
	CTL clusters (n=188)	1.57 ± 0.59	1.63 ± 1.14	1.60 ± 1.07	1.42 ± 3.16	1.79 ± 1.56	1.69 ± 1.33	1.72 ± 1.54	1.33 ± 1.27
	Single cis-eQTL regions (n=232)	1.5±0.67	1.61±1.33	1.59±1.18	1.26±2.11	1.58±1.32	1.7±1.65	1.66±1.56	1.4±1.3
	Random regions (n=500)	1 ± 0.84	1 ± 1.42	1 ± 1.26	1 ± 3.44	1 ± 1.44	1 ± 1.47	1 ± 1.48	1 ± 1.31
**Anova P values**		1.88E-41	1.06E-30	9.36E-15	0.0081	5.40E-13	6.99E-25	6.61E-22	1.34E-07
**Post-hoc Tukey P values**	Cis-eqtl clusters vs CTL	<e-5	<e-5	0.12	0.099	0.69	<e-5	<e-5	0.0094
	Cis-eqtl clusters vs single cis-eQTL regions	<e-5	<e-5	0.1	0.04	0.195	<e-5	<e-5	0.02
	Cis-eqtl clusters vs random	<e-5	<e-5	<e-5	0.0063	4.00E-05	<e-5	<e-5	1.00E-05
	Control and single cis-eQTL regions	0.76	0.99	0.99	0.95	0.45	0.99	0.979	0.94
	CTL vs random	<e-5	<e-5	<e-5	0.41	0<e-5	<e-5	<e-5	0.019
	Random vs single cis-eQTL regions	<e-5	<e-5	<e-5	0.73	<e-5	<e-5	<e-5	8.00E-04
	Cis-eqtl clusters vs CTL	<e-5	<e-5	0.12	0.099	0.690	<e-5	<e-5	0.009

		CTSF	h3ac	gata4	MEF2A	NKX2_5	SRF	TBX5	P300
**Size of flanking regions**	1 MB								
**Normalized abundance of TFs / MB (mean ± SD)**	Cis-eqtl clusters (n=42)	2.15 ± 0.7	3.16 ± 1.98	1.87 ± 1.18	2.63 ± 5.5	1.82 ± 1.43	2.99 ± 2.2	2.90 ± 1.94	1.69 ± 1.11
	CTL clusters (n=188)	1.45 ± 0.56	1.48 ± 0.97	1.45 ± 0.87	1.38 ± 3.2	1.59 ± 1.33	1.51 ± 1.19	1.53 ± 1.35	1.23 ± 0.96
	Single cis-eQTL regions (n=232)	1.4±0.6	1.44±1.12	1.44±0.96	1.16±1.71	1.45±1.13	1.49±1.35	1.47±1.34	1.29±0.95
	Random regions (n=500)	1 ± 0.76	1 ± 1.27	1 ± 1.01	1 ± 2.92	1 ± 1.17	1 ± 1.32	1 ± 1.34	1 ± 1.08
**Anova P values**		4.82E-33	4.59E-27	7.69E-14	0.004	6.77E-11	1.98E-20	2.12E-18	5.31E-06
**Post-hoc Tukey P values**	Cis-eqtl clusters vs CTL	<e-5	<e-5	0.05	0.05	0.65	<e-5	<e-5	0.038
	Cis-eqtl clusters vs single cis-eQTL regions	<e-5	<e-5	0.04	0.013	0.25	<e-5	<e-5	0.087
	Cis-eqtl clusters vs random	<e-5	<e-5	<e-5	0.0028	0.00014	<e-5	<e-5	0.00017
	Control and single cis-eQTL regions	0.82	0.97	0.99	0.86	0.65	0.99	0.97	0.92
	CTL vs random	<e-5	3.00E-05	<e-5	0.42	<e-5	6.00E-05	4.00E-05	0.05
	Random vs single cis-eQTL regions	<e-5	5.00E-05	<e-5	0.9	2.00E-05	4.00E-05	9.00E-05	0.002
	Cis-eqtl clusters vs CTL	<e-5	<e-5	0.059	0.056	0.65	<e-5	<e-5	0.038

Table S8 Comparisons for respective normalized abundance of binding sites for regulatory factors in several sizes of genomic regions around "500 kB" clusters. Normalized abundances of binding sites for regulatory factors was calculated in regions containing cis-eQTL cluster, control cluster, single cis-eQTL region or random regions, and augmented by flanking region of either 250, 500, 1000 kb.

		CTSF	h3ac	gata4	MEF2A	NKX2_5	SRF	TBX5	P300
**Size of flanking regions**	250 kB								
**Normalized abundance of TFs / MB (mean ± SD)**	Cis-eqtl clusters (n=53)	2.50 ± 1.01	3.60 ± 2.53	1.96 ± 1.44	2.15 ± 4.14	1.91 ± 1.5	3.30 ± 2.59	3.18 ± 2.31	2.22 ± 1.81
	CTL clusters (n=59)	1.49 ± 0.56	1.53 ± 1.06	1.47 ± 1.37	1.30 ± 2.43	1.45 ± 1.27	1.37 ± 1.23	1.20 ± 0.94	1.31 ± 1.42
	Single cis-eQTL regions (n=232)	1.58±0.71	1.7±1.45	1.63±1.29	1.27±2.21	1.62±1.39	1.81±1.75	1.75±1.63	1.53±1.52
	Random regions (n=500)	1 ± 0.86	1 ± 1.47	1 ± 1.34	1 ± 3.71	1 ± 1.55	1 ± 1.51	1 ± 1.52	1 ± 1.41
**Anova P values**		6.89E-41	2.25E-30	3.74E-11	0.1	1.80E-08	3.74E-23	2.03E-22	1.15E-09
**Post-hoc Tukey P values**	Cis-eqtl clusters vs CTL	<e-5	<e-5	0.21	0.53	0.35	<e-5	<e-5	0.0062
	Cis-eqtl clusters vs single cis-eQTL regions	<e-5	<e-5	0.37	0.30	0.56	<e-5	<e-5	0.011
	Cis-eqtl clusters vs random	<e-5	<e-5	<e-5	0.079	0.00014	<e-5	<e-5	<e-5
	Control and single cis-eQTL regions	0.86	0.86	0.83	0.99	0.86	0.25	0.08	0.73
	CTL vs random	1.00E-04	0.058	0.053	0.91	0.12	0.35	0.79	0.42
	Random vs single cis-eQTL regions	<e-5	<e-5	<e-5	0.74	<e-5	<e-5	<e-5	4.00E-05
	Cis-eqtl clusters vs CTL	<e-5	<e-5	0.21	0.53	0.35	<e-5	<e-5	0.0062

		CTSF	h3ac	gata4	MEF2A	NKX2_5	SRF	TBX5	P300
**Size of flanking regions**	500 kB								
**Normalized abundance of TFs / MB (mean ± SD)**	Cis-eqtl clusters (n=53)	2.21 ± 0.78	3.08 ± 2.2	1.79 ± 1.34	2.19 ± 4.77	1.72 ± 1.35	2.82 ± 2.36	2.81 ± 2.03	1.87 ± 1.38
	CTL clusters (n=59)	1.26 ± 0.45	1.15 ± 0.75	1.30 ± 1.1	1.23 ± 2.17	1.30 ± 1.26	1.11 ± 0.95	0.99 ± 0.79	1.07 ± 1.05
	Single cis-eQTL regions (n=232)	1.46 ±0.66	1.56 ±1.26	1.54 ±1.1	1.25 ±1.99	1.52 ±1.24	1.62 ±1.54	1.59 ±1.49	1.33 ±1.12
	Random regions (n=500)	1 ± 0.81	1 ± 1.36	1 ± 1.16	1 ± 3.21	1 ± 1.33	1 ± 1.42	1 ± 1.44	1 ± 1.2
**Anova P values**		2.53E-31	1.46E-24	2.40E-10	0.0467	1.93E-07	1.39E-17	2.76E-18	4.06E-07
**Post-hoc Tukey P values**	Cis-eqtl clusters vs CTL	<e-5	<e-5	0.10	0.32	0.33	<e-5	<e-5	0.0022
	Cis-eqtl clusters vs single cis-eQTL regions	<e-5	<e-5	0.45	0.165	0.76	<e-5	<e-5	0.016
	Cis-eqtl clusters vs random	<e-5	<e-5	1.00E-05	0.03	0.0008	<e-5	<e-5	<e-5
	Control and single cis-eQTL regions	0.28507	0.18261	0.51	0.99	0.65	0.094	0.025	0.43
	CTL vs random	0.05502	0.84625	0.22	0.94	0.32	0.94	0.99	0.97
	Random vs single cis-eQTL regions	<e-5	<e-5	<e-5	0.71	<e-5	<e-5	<e-5	0.002
	Cis-eqtl clusters vs CTL	<e-5	<e-5	0.1	0.32	0.33	<e-5	<e-5	0.002

		CTSF	h3ac	gata4	MEF2A	NKX2_5	SRF	TBX5	P300
**Size of flanking regions**	1 MB								
**Normalized abundance of TFs / MB (mean ± SD)**	Cis-eqtl clusters (n=53)	1.97 ± 0.65	2.61 ±1.79	1.64 ± 1.03	1.97 ± 4.7	1.53 ± 1.15	2.43 ± 1.9	2.48 ± 1.79	1.69 ± 1.06
	CTL clusters (n=59)	1.14 ± 0.47	1.02 ± 0.66	1.16 ± 0.81	0.96 ± 1.59	1.16 ± 0.97	0.96 ± 0.77	0.88 ± 0.67	1.02 ± 0.2
	Single cis-eQTL regions (n=232)	1.38±0.6	1.42±1.1	1.42±0.91	1.1±1.58	1.42±1.07	1.46±1.32	1.45±1.32	1.3±0.95
	Random regions (n=500)	1 ± 0.74	1 ± 1.27	1 ± 0.97	1 ± 3.22	1 ± 1.15	1 ± 1.3	1 ± 1.32	1 ± 1.06
**Anova P values**		2.61E-25	1.28E-18	1.80E-09	0.13	3.93E-06	4.28E-14	3.91E-15	6.14E-07
**Post-hoc Tukey P values**	Cis-eqtl clusters vs CTL	<e-5	<e-5	0.042	0.25	0.28	<e-5	<e-5	0.0025
	Cis-eqtl clusters vs single cis-eQTL regions	<e-5	<e-5	0.43	0.19	0.9	1.00E-05	<e-5	0.055
	Cis-eqtl clusters vs random	<e-5	<e-5	2.00E-05	0.092	0.0056	<e-5	<e-5	1.00E-05
	Control and single cis-eQTL regions	0.087	0.12	0.25	0.98	0.38	0.04	0.016	0.21
	CTL vs random	0.41	0.99	0.59	0.99	0.73	0.99	0.91	0.99
	Random vs single cis-eQTL regions	<e-5	0.00014	<e-5	0.97	2.00E-05	9.00E-05	1.00E-04	0.0009
	Cis-eqtl clusters vs CTL	<e-5	<e-5	0.04	0.25	0.28	<e-5	<e-5	0.0025

Table S9 Descriptive information concerning the 42 "250 kB" cis-eQTL clusters

Chr	*Start*	end	Length	Number of cis-eQTsL	Symbols of cis-eQTL genes
1	*172981698*	173434853	453155	11	FCGR3,1700009P17RIK,SDHCAPOA2, FCER1G,B4GALT3,PPOX,UFC1,KLHDC9,F11R,REFBP2
1	*173934520*	174206100	271580	5	VANGL2,NCSTN,COPA,PEX19,ATP1A2
1	*182837968*	183096385	258417	4	PYCR2,LEFTY1,TMEM63A,CNIH4
2	*25207840*	25354300	146460	4	DPP7,UAP1L1,ENTPD2,C8G
2	*103921214*	103964818	43604	3	CD59B,CD59A,A930018P22RIK
2	*152590596*	153048274	457678	6	COX4I2,FKHL18,PDRG1,BC020535,TM9SF4,TSPYL3
3	*35794225*	35988326	194101	3	LOC100046841,MCCC1,ACAD9
3	*87719934*	87861011	141077	3	HDGF,NES,APOA1BP,
4	*41585069*	41791875	206806	3	DNAIC1,CCL27,CCL19,
4	*62160379*	62363247	202868	3	HDHD3,ALAD,RGS3,
4	*129193683*	129525601	331918	4	LOC100046039,X2510006D16RIK,CCDC28B,PTP4A2
4	*132086525*	132456695	370170	4	ATPIF1,EYA3,XKR8,BC008163
4	*132754041*	133147532	393491	7	WASF2,MAP3K6SLC9A1,4732473B16RIK, 2300002D11RIK,NUDCGPN2,
4	*133912418*	134093953	181535	4	EXTL1,STMN1,2410166I05RIK,SEPN1
4	*155207073*	155364772	157699	6	AURKAIP1,DVL1,ACAP3,LOC545056,FAM132A,B3GALT6,
5	*147765528*	147890516	124988	3	GTF3A,MTIF3,POLR1D
6	*126875000*	127079686	204686	3	RAD51AP1,X9630033F20RIK,CCND2
6	*145123111*	145168681	45570	4	LRMP,CASC1,LYRM5,KRAS
7	*19441998*	19831693	389695	4	MILL2,IRF2BP1,DMWD,D630048P19RIK
7	*30975506*	31426230	450724	5	CAPNS1,TBCB,TYROBP,HSPB6,RBM42
7	*87461806*	87505819	44013	4	RCCD1,LOC675567,UNC45A,MAN2A2
8	*32207411*	32371718	164307	3	DUSP26,RBM13,FUT10
8	*87194527*	87621384	426857	11	NACC1,TRMT1,NFIX,FARSA, GCDH,PRDX2,HOOK2,ASNA1, DHPS,1500041N16RIK,MAN2B1
8	*124990160*	125154233	164073	3	RNF166,TRAPPC2L,CBFA2T3H
9	*34933919*	35024383	90464	3	DCPS,FOXRED1,SRPR
9	*44199978*	44212071	12093	3	HYOU1,SLC37A4,TRAPPC4
9	*44806893*	44962140	155247	3	CD3E,AMICA1,SCN4B
9	*106097518*	106372827	275309	7	PPM1M,TWF2,DUSP7,RPL29,ACY1,ABHD14A,PARP3
11	*58804677*	59235079	430402	4	TRIM11,GJC2,MRPL55,1110031B06RIK
11	*59589470*	59689992	100522	3	MPRIP,COPS3,NT5M
11	*82619501*	82756537	137036	3	RFFL,LOC100044934,UNC45B
11	*94830377*	95136060	305683	5	SGCA,SAMD14,PDK2,ITGA3,MYST2
11	*96799199*	96910230	111031	3	PNPO,SCRN2,MRPL10
11	*114844007*	115044431	200424	3	4732429D16RIK,SLC9A3R1,LOC100044159
13	*64234749*	64540754	306005	6	ZFP367,HABP4,CDC14B,1110018J18RIK, CTSL,CCRK
13	*113657734*	114008245	350511	5	PPAP2A,SKIV2L2,GPX8,2310016C16RIK, ESM1
15	*76545807*	76682760	136953	3	LRRC24,C030006K11RIK,ZFP251
15	*85727285*	85967913	240628	3	TRMU,CELSR1,GRAMD4
16	*20651876*	20742404	90528	3	PSMD2,EIF4G1,CHRD
17	*24645834*	25029013	383179	5	TRAF7,GFER,NDUFB10,FAHD1,SPSB3
17	*34056177*	34163052	106875	3	ZBTB22,WDR46,H2.KE6
18	*37847239*	37909257	62018	3	PCDHGA4,PCDHGB2,PCDHGA10

Table S10 Descriptive information concerning "control clusters"

Table S11 Descriptive information concerning "random boxes"

Tables S10 and S11 are available for download at http://www.g3journal.org/lookup/suppl/doi:10.1534/g3.113.005546/-/DC1.
