# Supplementary material for: Genome-Wide Detection of Gene Coexpression Domains Showing Linkage to Regions Enriched with Polymorphic Retrotransposons in Recombinant Inbred Mouse Strains
Source: G3 (Bethesda). 2013 Apr 1;3(4):597–605. doi: 10.1534/g3.113.005546 (PMC3618347; doi:10.1534/g3.113.005546)
Supplement: Supporting Information [file supp_g3.113.005546_FigureS1.pdf]

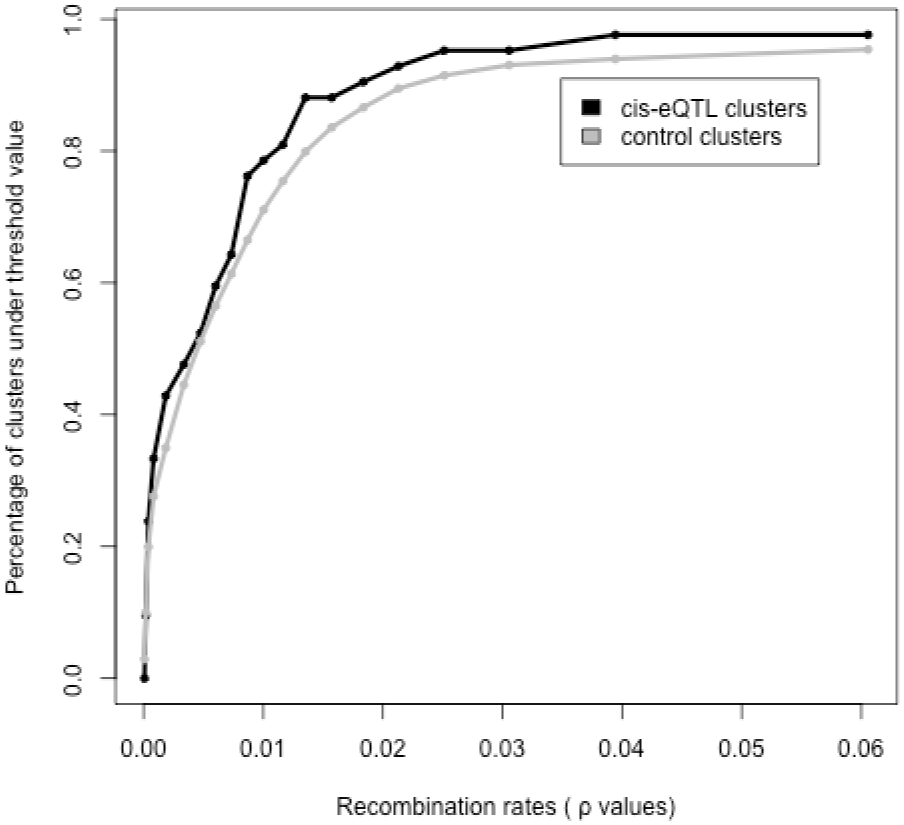

Figure S1 Distribution of recombination rates in regions corresponding to either cis-eQTL or control clusters. The recombination rate values corresponded to the ρ values, as calculated and reported by Brunschwig H et al. (Genetics 191: 757–764, 2012). According to the Chi-square test, there was no significant difference in the distributions of recombination rates in the 2 types of regions ($P > 0.3$).
