# Supplementary material for: Genome-Wide Detection of Gene Coexpression Domains Showing Linkage to Regions Enriched with Polymorphic Retrotransposons in Recombinant Inbred Mouse Strains
Source: G3 (Bethesda). 2013 Apr 1;3(4):597–605. doi: 10.1534/g3.113.005546 (PMC3618347; doi:10.1534/g3.113.005546)
Supplement: Supporting Information [file supp_g3.113.005546_TableS1.pdf]

Table S1 Abundance of polymorphic and total TEs in mouse genomes. Lists of polymorphic TEs were obtained from either the publication of Nellåker et al (2012) or from the MouseIndelDB database. Lists of fixed TEs were obtained from the Transpogene database. n/a: non available.

Database	Genetic origin	full LINEs	LINE frag.	LTR-TEs	SINEs
**Nellåker et al. (polymorphic TEs)**	Both strains	1808	2969	4734	4303
	C57(+) / A/J(-)	606	1015	1901	2378
	C57(-) / A/J(+)	1202	1954	2833	1925
**MouseIndelDB (polymorphic TEs)**	Both strains			2413	1512
	C57(+) / A/J(-)			1436	1512
	C57(-) / A/J(+)			977	0
**Transpogene (fixed TEs)**		78,002	n/a	84,724	190,057
