# Supplementary material for: Genome-Wide Detection of Gene Coexpression Domains Showing Linkage to Regions Enriched with Polymorphic Retrotransposons in Recombinant Inbred Mouse Strains
Source: G3 (Bethesda). 2013 Apr 1;3(4):597–605. doi: 10.1534/g3.113.005546 (PMC3618347; doi:10.1534/g3.113.005546)
Supplement: Supporting Information [file supp_g3.113.005546_TableS2.pdf]

Table S2 Summary of gene expression datasets from mouse RIS. Each line provides information about which tissue and which RIS panel was used for each gene expression dataset, as well as about the number of strains that were profiled and the nature of the microarray platform. The GN access numbers corresponds to the identification number of corresponding datasets in GeneNetwork.

Tissue	RIS panel	# of strains used	Microarray platform	GN access #
Eye	AxB/BxA	26	Illumina MouseRef-6	GN210
Eye	BxD	68	Affymetrix Mouse Genome 430	GN207
Kidney	BxD	54	Affymetrix Mouse Genome 430	GN240
Hippocampus	BxD	67	Affymetrix Mouse Genome 430	GN112
Hypothalamus	BxD	33	Affymetrix MoGene 1.0 ST	GN281
Cerebellum	BxD	28	Affymetrix Mouse Genome 430	GN72
