# Supplementary material for: Genome-Wide Detection of Gene Coexpression Domains Showing Linkage to Regions Enriched with Polymorphic Retrotransposons in Recombinant Inbred Mouse Strains
Source: G3 (Bethesda). 2013 Apr 1;3(4):597–605. doi: 10.1534/g3.113.005546 (PMC3618347; doi:10.1534/g3.113.005546)
Supplement: Supporting Information [file supp_g3.113.005546_TableS3.pdf]

Table S3 Properties of cis-eQTL and control clusters (defined using three different window sizes). In addition to the sizes of the intervals between detected genes, some additional criteria were used in the selection of control clusters in order to have them match cis-eQTL clusters for size and density of total genes. For the 250 kB intervals, only clusters <400 kB and containing more than 3 genes were kept; for the 500 kB, only clusters <650 kB and containing at least 6 genes were kept; for the 750 kB intervals, only clusters of <800 kb and containing at least 8 genes were kept.

**Maximum interval between cis-eQTLs or detected genes**	**250kB**			**500kB**			**750kB**		
	Cis-eQTL clusters	Control cluters	p-val	Cis-eQTL clusters	Control cluters	p-val	Cis-eQTL clusters	Control cluters	p-val
Size of boxes (in kb)	221.9 ±130	248±	0.2	467.1 ±486	456±119	0.87	658.8 ±553	561±123	0.2
Number of detected genes	4.2 ±1.9	4.75±	0.1	4.9 ±3.5	5.23±2.46	0.63	5.1 ±3.6	6.6±3.89	0.11
Total number of genes	7.2 ±6.6	6.30 ±2.9	0.38	12.3 ±16	10.1±3.45	0.35	15.70±17.6	14.9±11.1	0.81
Number of clusters	42	188		53	59		61	21	
