# Supplementary material for: Genome-Wide Detection of Gene Coexpression Domains Showing Linkage to Regions Enriched with Polymorphic Retrotransposons in Recombinant Inbred Mouse Strains
Source: G3 (Bethesda). 2013 Apr 1;3(4):597–605. doi: 10.1534/g3.113.005546 (PMC3618347; doi:10.1534/g3.113.005546)
Supplement: Supporting Information [file supp_g3.113.005546_TableS4.pdf]

Table S4 Normalized abundance of polymorphic and fixed TEs in several sizes of genomic regions around "250 kB" clusters. Normalized abundance of TEs was calculated by dividing the abundance of each element with that found in random regions. Test regions corresponded to those containing cis-eQTLs (either clustered or single) or control clusters, each region being augmented by flanking regions of either 250, 500, 1000 kb.

		Polymorphic				Fixed	
		full LINEs	LINE frag.	LTR-TEs	SINEs	LTR-TEs	SINEs
**Size of flanking regions**	250 kB						
**Normalized abundance of TEs / MB (mean ± SD)**	Cis-eqtl clusters (n=42)	0.51 ± 1.39	0.77 ± 1.42	2.27 ± 1.75	4.78 ± 3.51	1.55 ± 2.17	2.85 ± 3.01
	CTL clusters (n=188)	0.46 ± 1.16	0.55 ± 1.01	1.28 ± 1.58	1.45 ± 2.07	1.53 ± 1.3	2.05 ± 1.62
	Single cis-eQTL regions (n=232)	0.59 ± 1.32	0.94 ± 1.33	1.8± 1.53	1.97±1.84	1.53±1.26	1.71±1.78
	Random regions (n=500)	1 ± 2.04	1 ± 1.72	1 ± 1.39	1 ± 1.72	1 ± 1.27	1 ± 1.42
**Anova P values**		0.00039	0.0059	1.22E-13	1.11E-33	3.15E-08	4.40E-20
**Post-hoc Tukey P values**	Cis-eqtl clusters vs CTL	0.99	0.83	0.00053	<e-5	0.99	0.02306
	Cis-eqtl clusters vs single cis-eQTL regions	0.99	0.90	0.22	<e-5	0.99	0.00023
	Cis-eqtl clusters vs random	0.28	0.77	<e-5	<e-5	0.048	<e-5
	Control and single cis-eQTL regions	0.8	0.042	0.0019	0.029	0.99	0.15
	CTL vs random	0.001	0.003	0.12	0.03	2.00E-05	<e-5
	Random vs single cis-eQTL regions	0.015	0.96	<e-5	<e-5	<e-5	<e-5

		Polymorphic				Fixed	
		full LINEs	LINE frag.	LTR-TEs	SINEs	LTR-TEs	SINEs
**Size of flanking regions**	500 kB						
**Normalized abundance of TEs / MB (mean ± SD)**	Cis-eqtl clusters (n=42)	0.54 ± 1.42	0.98 ± 1.69	1.88 ± 1.43	4.14 ± 2.84	1.39 ± 1.22	2.46 ± 1.86
	CTL clusters (n=188)	0.61 ± 1.16	0.65 ± 1.05	1.10 ± 1.25	1.27 ± 1.59	1.41 ± 1.10	1.79 ± 1.39
	Single cis-eQTL regions (n=232)	0.75±1.24	0.93±1.07	1.62±1.21	1.7±1.45	1.39±1.1	1.53±1.48
	Random regions (n=500)	1 ± 1.77	1 ± 1.51	1 ± 1.20	1 ± 1.6	1 ± 1.10	1 ± 1.29
**Anova P values**		0.0068	0.024	1.12E-11	2.22E-31	1.01E-06	6.32E-17
**Post-hoc Tukey P values**	Cis-eqtl clusters vs CTL	0.99	0.47	0.001	<e-5	0.99	0.024
	Cis-eqtl clusters vs single cis-eQTL regions	0.83	0.99	0.58	<e-5	1	0.00041
	Cis-eqtl clusters vs random	0.23	0.99	5.00E-05	<e-5	0.12352	<e-5
	Control and single cis-eQTL regions	0.75	0.13	8.00E-05	0.03	0.99	0.22
	CTL vs random	0.014	0.013	0.79	0.21	1.00E-04	<e-5
	Random vs single cis-eQTL regions	0.18	0.92	<e-5	<e-5	6.00E-05	1.00E-05

		Polymorphic				Fixed	
		**full LINEs**	**LINE frag.**	**LTR-TEs**	**SINEs**	**LTR-TEs**	**SINEs**
**Size of flanking regions**	1 MB						
**Normalized abundance of TEs / MB (mean ± SD**	Cis-eqtl clusters (n=42)	0.59 ± 1.25	0.93 ± 1.27	1.71 ± 1.17	3.47 ± 2.36	1.26 ± 0.93	2.12 ± 1.54
	CTL clusters (n=188)	0.76 ± 1.26	0.72 ± 1.02	1.06 ± 0.92	1.14 ± 1.17	1.33 ± 0.99	1.53 ± 1.1
	Single cis-eQTL regions (n=232)	0.48±1.71	0.81±1.91	1.76±2.28	2.69±3.3	1.7±1.58	2.02±2.02
	Random regions (n=500)	1 ± 1.47	1 ± 1.39	1 ± 1.03	1 ± 1.39	1 ± 0.87	1 ± 1.11
**Anova P values**		0.00013	0.1	4.95E-11	7.17E-31	1.75E-13	3.84E-20
**Post-hoc Tukey P values**	Cis-eqtl clusters vs CTL	0.91	0.82	0.037	<e-5	0.98	0.069
	Cis-eqtl clusters vs single cis-eQTL regions	0.96	0.96	0.99	0.1	0.083	0.97
	Cis-eqtl clusters vs random	0.32	0.99	0.011	<e-5	0.46	1.00E-05
	Control and single cis-eQTL regions	0.22	0.91	<e-5	<e-5	0.0034	0.0024
	CTL vs random	0.22	0.11	0.96	0.86	0.0034	6.00E-05
	Random vs single cis-eQTL regions	7.00E-05	0.37	<e-5	<e-5	<e-5	<e-5
