# Supplementary material for: Genome-Wide Detection of Gene Coexpression Domains Showing Linkage to Regions Enriched with Polymorphic Retrotransposons in Recombinant Inbred Mouse Strains
Source: G3 (Bethesda). 2013 Apr 1;3(4):597–605. doi: 10.1534/g3.113.005546 (PMC3618347; doi:10.1534/g3.113.005546)
Supplement: Supporting Information [file supp_g3.113.005546_TableS6.pdf]

Table S6 Most significantly enriched binding sites in polymorphic SINEs

Motif Name (family)	CHIP-Seq cell line	Consensus	P-value	% of Target Sequences with Motif	% of Background Sequences with Motif
**CTCFL /BORIS (Zinc finger)**	K562	CNNBRGCGCCCCCTGSTGGC	1e-1283	63.96%	3.33%
ZNF143 (Zinc finger)	CUTTL	ATTTCCCAGVAKSCY	1e-1273	73.63%	5.91%
c-Myc	mES	VVCCACGTGG	1e-1213	75.98%	7.34%
n-Myc (Helix-Loop-Helix)	mES	VRCCACGTGG	1e-1116	76.39%	8.77%
Max (Helix-Loop-Helix))	K562	RCCACGTGGYYN	1e-1094	77.54%	9.57%
RUNX (Runt)	HPC7	SAAACCACAG	1e-1000	76.44%	10.61%
Gfi1b (Zinc finger)	HPC7	MAATCACTGC	1e-996	72.28%	8.82%
RUNX2 (Runt)	PCa	NWAACCACADNN	1e-906	76.44%	12.37%
RUNX1 (Runt)	Jurkat	AAACCACARM	1e-820	76.70%	14.39%
Cdx2 (Homeobox)	mES	GYMATAAAAH	1e-623	63.70%	11.95%
Olig2 (Helix-Loop-Helix))	Neuron	RCCATMTGTT	1e-613	79.10%	22.14%
HNF4a (Nuclear Receptors)	HpeG2	CARRGKBCAAAGTYCA	1e-359	33.54%	4.58%
GATA3 (Zinc finger)	iTreg	AGATSTNDNNDSAGATAASN	1e-322	23.19%	1.97%
Erra(NR)	HepG2	CAAAGGTCAG	1.00E-304	62.35%	22.53%
FOXA1 (Forkhead)	MCF7	WAAGTAAACA	1.00E-304	53.46%	16.26%
Smad3 (MAD)	NPC	TWGTCTGV	1.00E-265	68.28%	29.69%
FOXA1 (Forkhead)	LNCaP	WAAGTAAACA	1.00E-265	54.08%	18.54%
AR-halfsite (Nuclear Receptors)	LNCaP	CCAGGAACAG	1.00E-232	78.00%	41.54%
Nkx2.5 (Homeobox)	HL1	RRSCACTYAA	1.00E-182	56.89%	25.78%
Nanog (Homeobox)	mES	RGCCATTAAC	1.00E-106	69.53%	44.63%
NeuroD1 (Helix-Loop-Helix)	Islet	GCCATCTGTT	1.00E-102	25.07%	8.50%
**CTCF (Zinc finger)**	CD4+	AYAGTGCCMYCTRGTGGCCA	1.00E-67	9.26%	1.82%
