# Supplementary material for: Genome-Wide Detection of Gene Coexpression Domains Showing Linkage to Regions Enriched with Polymorphic Retrotransposons in Recombinant Inbred Mouse Strains
Source: G3 (Bethesda). 2013 Apr 1;3(4):597–605. doi: 10.1534/g3.113.005546 (PMC3618347; doi:10.1534/g3.113.005546)
Supplement: Supporting Information [file supp_g3.113.005546_TableS8.pdf]

Table S8 Comparisons for respective normalized abundance of binding sites for regulatory factors in several sizes of genomic regions around "500 kB" clusters. Normalized abundances of binding sites for regulatory factors was calculated in regions containing cis-eQTL cluster, control cluster, single cis-eQTL region or random regions, and augmented by flanking region of either 250, 500, 1000 kb.

		CTSF	h3ac	gata4	MEF2A	NKX2_5	SRF	TBX5	P300
**Size of flanking regions**	250 kB								
**Normalized abundance of TFs / MB (mean ± SD)**	Cis-eqtl clusters (n=53)	2.50 ± 1.01	3.60 ± 2.53	1.96 ± 1.44	2.15 ± 4.14	1.91 ± 1.5	3.30 ± 2.59	3.18 ± 2.31	2.22 ± 1.81
	CTL clusters (n=59)	1.49 ± 0.56	1.53 ± 1.06	1.47 ± 1.37	1.30 ± 2.43	1.45 ± 1.27	1.37 ± 1.23	1.20 ± 0.94	1.31 ± 1.42
	Single cis-eQTL regions (n=232)	1.58±0.71	1.7±1.45	1.63±1.29	1.27±2.21	1.62±1.39	1.81±1.75	1.75±1.63	1.53±1.52
	Random regions (n=500)	1 ± 0.86	1 ± 1.47	1 ± 1.34	1 ± 3.71	1 ± 1.55	1 ± 1.51	1 ± 1.52	1 ± 1.41
**Anova P values**		6.89E-41	2.25E-30	3.74E-11	0.1	1.80E-08	3.74E-23	2.03E-22	1.15E-09
**Post-hoc Tukey P values**	Cis-eqtl clusters vs CTL	<e-5	<e-5	0.21	0.53	0.35	<e-5	<e-5	0.0062
	Cis-eqtl clusters vs single cis-eQTL regions	<e-5	<e-5	0.37	0.30	0.56	<e-5	<e-5	0.011
	Cis-eqtl clusters vs random	<e-5	<e-5	<e-5	0.079	0.00014	<e-5	<e-5	<e-5
	Control and single cis-eQTL regions	0.86	0.86	0.83	0.99	0.86	0.25	0.08	0.73
	CTL vs random	1.00E-04	0.058	0.053	0.91	0.12	0.35	0.79	0.42
	Random vs single cis-eQTL regions	<e-5	<e-5	<e-5	0.74	<e-5	<e-5	<e-5	4.00E-05
	Cis-eqtl clusters vs CTL	<e-5	<e-5	0.21	0.53	0.35	<e-5	<e-5	0.0062

		CTSF	h3ac	gata4	MEF2A	NKX2_5	SRF	TBX5	P300
**Size of flanking regions**	500 kB								
**Normalized abundance of TFs / MB (mean ± SD)**	Cis-eqtl clusters (n=53)	2.21 ± 0.78	3.08 ± 2.2	1.79 ± 1.34	2.19 ± 4.77	1.72 ± 1.35	2.82 ± 2.36	2.81 ± 2.03	1.87 ± 1.38
	CTL clusters (n=59)	1.26 ± 0.45	1.15 ± 0.75	1.30 ± 1.1	1.23 ± 2.17	1.30 ± 1.26	1.11 ± 0.95	0.99 ± 0.79	1.07 ± 1.05
	Single cis-eQTL regions (n=232)	1.46 ±0.66	1.56 ±1.26	1.54 ±1.1	1.25 ±1.99	1.52 ±1.24	1.62 ±1.54	1.59 ±1.49	1.33 ±1.12
	Random regions (n=500)	1 ± 0.81	1 ± 1.36	1 ± 1.16	1 ± 3.21	1 ± 1.33	1 ± 1.42	1 ± 1.44	1 ± 1.2
**Anova P values**		2.53E-31	1.46E-24	2.40E-10	0.0467	1.93E-07	1.39E-17	2.76E-18	4.06E-07
**Post-hoc Tukey P values**	Cis-eqtl clusters vs CTL	<e-5	<e-5	0.10	0.32	0.33	<e-5	<e-5	0.0022
	Cis-eqtl clusters vs single cis-eQTL regions	<e-5	<e-5	0.45	0.165	0.76	<e-5	<e-5	0.016
	Cis-eqtl clusters vs random	<e-5	<e-5	1.00E-05	0.03	0.0008	<e-5	<e-5	<e-5
	Control and single cis-eQTL regions	0.28507	0.18261	0.51	0.99	0.65	0.094	0.025	0.43
	CTL vs random	0.05502	0.84625	0.22	0.94	0.32	0.94	0.99	0.97
	Random vs single cis-eQTL regions	<e-5	<e-5	<e-5	0.71	<e-5	<e-5	<e-5	0.002
	Cis-eqtl clusters vs CTL	<e-5	<e-5	0.1	0.32	0.33	<e-5	<e-5	0.002

		CTSF	h3ac	gata4	MEF2A	NKX2_5	SRF	TBX5	P300
**Size of flanking regions**	1 MB								
**Normalized abundance of TFs / MB (mean ± SD)**	Cis-eqtl clusters (n=53)	1.97 ± 0.65	2.61 ±1.79	1.64 ± 1.03	1.97 ± 4.7	1.53 ± 1.15	2.43 ± 1.9	2.48 ± 1.79	1.69 ± 1.06
	CTL clusters (n=59)	1.14 ± 0.47	1.02 ± 0.66	1.16 ± 0.81	0.96 ± 1.59	1.16 ± 0.97	0.96 ± 0.77	0.88 ± 0.67	1.02 ± 0.2
	Single cis-eQTL regions (n=232)	1.38±0.6	1.42±1.1	1.42±0.91	1.1±1.58	1.42±1.07	1.46±1.32	1.45±1.32	1.3±0.95
	Random regions (n=500)	1 ± 0.74	1 ± 1.27	1 ± 0.97	1 ± 3.22	1 ± 1.15	1 ± 1.3	1 ± 1.32	1 ± 1.06
**Anova P values**		2.61E-25	1.28E-18	1.80E-09	0.13	3.93E-06	4.28E-14	3.91E-15	6.14E-07
**Post-hoc Tukey P values**	Cis-eqtl clusters vs CTL	<e-5	<e-5	0.042	0.25	0.28	<e-5	<e-5	0.0025
	Cis-eqtl clusters vs single cis-eQTL regions	<e-5	<e-5	0.43	0.19	0.9	1.00E-05	<e-5	0.055
	Cis-eqtl clusters vs random	<e-5	<e-5	2.00E-05	0.092	0.0056	<e-5	<e-5	1.00E-05
	Control and single cis-eQTL regions	0.087	0.12	0.25	0.98	0.38	0.04	0.016	0.21
	CTL vs random	0.41	0.99	0.59	0.99	0.73	0.99	0.91	0.99
	Random vs single cis-eQTL regions	<e-5	0.00014	<e-5	0.97	2.00E-05	9.00E-05	1.00E-04	0.0009
	Cis-eqtl clusters vs CTL	<e-5	<e-5	0.04	0.25	0.28	<e-5	<e-5	0.0025
