# Supplementary material for: Genome-Wide Detection of Gene Coexpression Domains Showing Linkage to Regions Enriched with Polymorphic Retrotransposons in Recombinant Inbred Mouse Strains
Source: G3 (Bethesda). 2013 Apr 1;3(4):597–605. doi: 10.1534/g3.113.005546 (PMC3618347; doi:10.1534/g3.113.005546)
Supplement: Supporting Information [file supp_g3.113.005546_TableS9.pdf]

Table S9 Descriptive information concerning the 42 "250 kB" cis-eQTL clusters

Chr	*Start*	end	Length	Number of cis-eQTsL	Symbols of cis-eQTL genes
1	*172981698*	173434853	453155	11	FCGR3,1700009P17RIK,SDHCAPOA2, FCER1G,B4GALT3,PPOX,UFC1,KLHDC9,F11R,REFBP2
1	*173934520*	174206100	271580	5	VANGL2,NCSTN,COPA,PEX19,ATP1A2
1	*182837968*	183096385	258417	4	PYCR2,LEFTY1,TMEM63A,CNIH4
2	*25207840*	25354300	146460	4	DPP7,UAP1L1,ENTPD2,C8G
2	*103921214*	103964818	43604	3	CD59B,CD59A,A930018P22RIK
2	*152590596*	153048274	457678	6	COX4I2,FKHL18,PDRG1,BC020535,TM9SF4,TSPYL3
3	*35794225*	35988326	194101	3	LOC100046841,MCCC1,ACAD9
3	*87719934*	87861011	141077	3	HDGF,NES,APOA1BP,
4	*41585069*	41791875	206806	3	DNAIC1,CCL27,CCL19,
4	*62160379*	62363247	202868	3	HDHD3,ALAD,RGS3,
4	*129193683*	129525601	331918	4	LOC100046039,X2510006D16RIK,CCDC28B,PTP4A2
4	*132086525*	132456695	370170	4	ATPIF1,EYA3,XKR8,BC008163
4	*132754041*	133147532	393491	7	WASF2,MAP3K6SLC9A1,4732473B16RIK, 2300002D11RIK,NUDCGPN2,
4	*133912418*	134093953	181535	4	EXTL1,STMN1,2410166I05RIK,SEPN1
4	*155207073*	155364772	157699	6	AURKAIP1,DVL1,ACAP3,LOC545056,FAM132A,B3GALT6,
5	*147765528*	147890516	124988	3	GTF3A,MTIF3,POLR1D
6	*126875000*	127079686	204686	3	RAD51AP1,X9630033F20RIK,CCND2
6	*145123111*	145168681	45570	4	LRMP,CASC1,LYRM5,KRAS
7	*19441998*	19831693	389695	4	MILL2,IRF2BP1,DMWD,D630048P19RIK
7	*30975506*	31426230	450724	5	CAPNS1,TBCB,TYROBP,HSPB6,RBM42
7	*87461806*	87505819	44013	4	RCCD1,LOC675567,UNC45A,MAN2A2
8	*32207411*	32371718	164307	3	DUSP26,RBM13,FUT10
8	*87194527*	87621384	426857	11	NACC1,TRMT1,NFIX,FARSA, GCDH,PRDX2,HOOK2,ASNA1, DHPS,1500041N16RIK,MAN2B1
8	*124990160*	125154233	164073	3	RNF166,TRAPPC2L,CBFA2T3H
9	*34933919*	35024383	90464	3	DCPS,FOXRED1,SRPR
9	*44199978*	44212071	12093	3	HYOU1,SLC37A4,TRAPPC4
9	*44806893*	44962140	155247	3	CD3E,AMICA1,SCN4B
9	*106097518*	106372827	275309	7	PPM1M,TWF2,DUSP7,RPL29,ACY1,ABHD14A,PARP3
11	*58804677*	59235079	430402	4	TRIM11,GJC2,MRPL55,1110031B06RIK
11	*59589470*	59689992	100522	3	MPRIP,COPS3,NT5M
11	*82619501*	82756537	137036	3	RFFL,LOC100044934,UNC45B
11	*94830377*	95136060	305683	5	SGCA,SAMD14,PDK2,ITGA3,MYST2
11	*96799199*	96910230	111031	3	PNPO,SCRN2,MRPL10
11	*114844007*	115044431	200424	3	4732429D16RIK,SLC9A3R1,LOC100044159
13	*64234749*	64540754	306005	6	ZFP367,HABP4,CDC14B,1110018J18RIK, CTSL,CCRK
13	*113657734*	114008245	350511	5	PPAP2A,SKIV2L2,GPX8,2310016C16RIK, ESM1
15	*76545807*	76682760	136953	3	LRRC24,C030006K11RIK,ZFP251
15	*85727285*	85967913	240628	3	TRMU,CELSR1,GRAMD4
16	*20651876*	20742404	90528	3	PSMD2,EIF4G1,CHRD
17	*24645834*	25029013	383179	5	TRAF7,GFER,NDUFB10,FAHD1,SPSB3
17	*34056177*	34163052	106875	3	ZBTB22,WDR46,H2.KE6
18	*37847239*	37909257	62018	3	PCDHGA4,PCDHGB2,PCDHGA10
